# Perceptions and Reasons Regarding E-Cigarette Use among Users and Non-Users: A Narrative Literature Review

**DOI:** 10.3390/ijerph15061190

**Published:** 2018-06-06

**Authors:** Kim A. G. J. Romijnders, Liesbeth van Osch, Hein de Vries, Reinskje Talhout

**Affiliations:** 1 Center for Health Protection (GZB), National Institute for Public Health and the Environment (RIVM), 3721 MA Bilthoven, The Netherlands; reinskje.talhout@rivm.nl; 2 Department of Health Promotion, School for Public Health and Primary Care (CAPHRI), Maastricht University, P.O. Box 616 6200 MD Maastricht, The Netherlands; liesbeth.vanosch@maastrichtuniversity.nl (L.v.O.); hein.devries@maastrichtuniversity.nl (H.d.V.)

**Keywords:** electronic cigarette, adults, youth, perceptions, reasons, dual use

## Abstract

This paper aims to provide an in-depth understanding of the attractiveness of e-cigarettes for several different groups. For this purpose, perceptions of and reasons for e-cigarette use were systematically reviewed as reported by e-cigarette users, cigarette smokers, dual users, and non-users, among both adults and youth. MEDLINE^®^ and Scopus were used to search for relevant articles, and references of included studies were also investigated. Two reviewers screened all titles and abstracts independently, blinded to authors and journal titles (Cohen’s Kappa = 0.83), resulting in 72 eligible articles. Risk perceptions, perceived benefits, and reasons for e-cigarette use were categorized in themes and sub-themes. Risk perceptions included harmfulness in general, and specific health risks. Perceived benefits included improved taste and smell, and safety for bystanders. Reasons for use included (health) benefits, curiosity, smoking cessation, and friends using e-cigarettes. The findings highlight that there is a variety of perceptions and reasons mentioned by adult and youth e-cigarette users, cigarette smokers, dual users, and non-users. As such, this overview provides valuable information for scientists, public health professionals, behavior change experts, and regulators to improve future research, risk communication, and possibilities to effectively regulate e-cigarettes.

## 1. Introduction 

Electronic Nicotine Delivery Systems (ENDS) are devices that vaporize a solution of nicotine, additives, glycerin, and propylene glycol that is inhaled by the user [1,2,3,4]. Electronic cigarettes are the most common type of ENDS. The variety of electronic cigarette (e-cigarette) devices available on the market is rapidly increasing. While early models mimic conventional cigarettes (in shape and size), newer models vary in product specifications (shape, size, battery, and tanks) [2,3,4,5,6]. In addition to product specifications, design and flavor characteristics are increasingly elaborate and appealing [7,8,9]. Research in recent years has demonstrated that the appeal of e-cigarettes has increased rapidly [2,3,4,5,6,10]. The prevalence of e-cigarette use is increasing, mostly among cigarette smokers, but recent research suggests that e-cigarette use is also increasing among non-smokers, and may even be a gateway to smoking [2,3,9,11,12,13,14,15,16,17,18,19,20,21,22]. Glasser et al. [3] noted that, regardless of smoking status, e-cigarettes are perceived as less harmful and addictive, and effective as a smoking cessation aid. Nevertheless, risk perceptions and perceived benefits for e-cigarette use might be different for e-cigarette users than non-users. Moreover, Pepper and Brewer [6] and Glasser et al. [3] indicated that reasons for e-cigarette use go beyond smoking cessation [23] among e-cigarette users. However, as the appeal of e-cigarettes is increasing among non-users, it is interesting to study the reasons non-users report that could lead them to initiate e-cigarette use, and whether these reasons differ from cigarette smokers switching to e-cigarettes. In order to better understand the process of switching from cigarettes to e-cigarettes or experimenting with e-cigarettes, it is important to have an insight into perceptions of e-cigarettes and reasons for use among different types of users [6,11,19,20,21,22,23,24,25,26]. This paper therefore provides an overview of such perceptions and reasons among adult and youth e-cigarette users, cigarette smokers, dual users, and non-users. 

The current overview provides scientists, public health professionals, behavior change experts, and regulators with key constructs for the development and validation of measures to assess perceptions of e-cigarettes and reasons for e-cigarette use. Public health professionals are able to use the overview on perceptions and reasons when developing health education and behavior change programs. On a population level, policy makers are able to use this inclusive overview to intensify smoking bans to avoid dual use and to target product characteristics of e-cigarettes attractive for specific user groups.

## 2. Materials and Methods 

### 2.1. Search

The search strategy developed for the purpose of this narrative review aimed to retrieve articles focusing on perceptions and reasons related to e-cigarette use without any restrictions on location. Databases searched (and interfaces) were MEDLINE (Ovid) and Scopus (without date restrictions) till February 2018. Concepts included in the search were “electronic cigarette”, “perception”, “reason”, “opinion”, and “smoking cessation” (see supplementary Table S1 for the full search strategy). The references of all included articles in our review were examined for additional references. To check the completeness of our search strategy, the final list of records was checked for inclusion of prior identified relevant research. 

### 2.2. Study Selection

**Figure 1:**
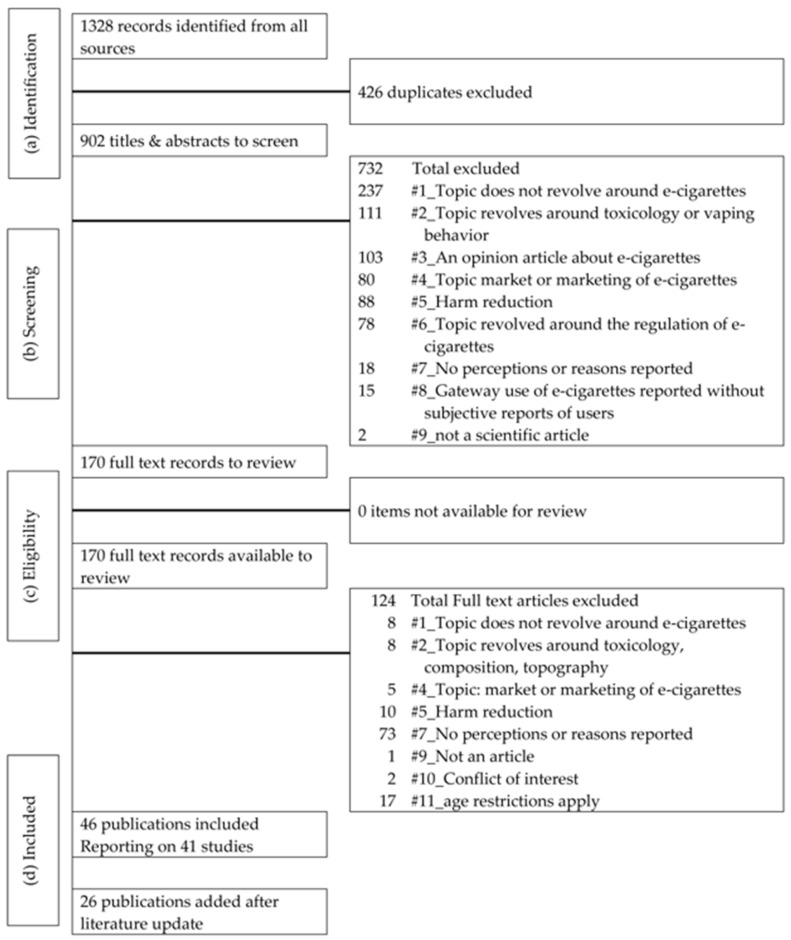
The Preferred Reporting Items for Systematic Reviews and Meta-analyses (PRISMA) guidelines. (a) Identification contains all records identified during the search. (b) Screening lists all reasons why articles were excluded based on title and abstract. (c) Eligibility records all the records available for full-text review. (d) Included reports all exclusion criteria used during full-text review.

Following the Preferred Reporting Items for Systematic Reviews and Meta-analyses (PRISMA) guidelines (see Figure 1. The PRISMA flow diagram), retrieved citations were screened, duplicates were eliminated, and the remaining citations were organized in EndNote [27]. Authors Kim A.G.J. Romijnders and Reinskje Talhout reviewed all titles using a previously agreed-upon exclusion criteria list (see supplementary Table S2). First, they independently screened a random sample of 86 titles and abstracts in which they were blinded to authors and journal titles, and reached strong agreement (Cohen’s Kappa = 0.83) [28]. Second, two authors (Kim A.G.J. Romijnders, Reinskje Talhout) independently screened all titles and abstracts, still blinded to authors and journal titles, using an Excel workbook designed specifically for screening [29]. Exclusion criteria were hierarchical in order, meaning that if the first exclusion criterion applies, the other exclusion criteria were not checked. Exclusion criteria were the following: (1) The article was not about e-cigarettes; (2) The article discussed toxicology and vaping behavior; (3) The article was an opinion piece; (4) The article discussed the market or marketing of e-cigarettes; (5) The article was about harm reduction; (6) The topic of the article was regulation; (7) The article did not include subjective reports; (8) The article described the gateway effect; or (9) It was not an article [27]. The full exclusion decision tree can be found in supplementary Table S2. Full-text articles were reviewed to determine final eligibility with the same exclusion decision tree (supplementary Table S2) [30], but two additional exclusion criteria applied: (10) Conflict of interest, and (11) Age restrictions. To make a distinction between adults (>18) and youth (<18), studies needed to apply clearly defined age restrictions (adults > 18 and youth < 18). An article was considered for inclusion if it was a quantitative or qualitative study focusing on subjective reports of participants, reporting on perceptions and/or reasons for e-cigarette use. The Excel workbooks are available upon request from the first author.

### 2.3. Data Extraction

Kim A.G.J. Romijnders extracted all relevant findings from the included studies (See Table S3). Due to the variety in research designs, it was not possible to generate a single quality score according to STROBE [31]. A single quality score, generated by the STROBE checklist, would limit the scope of this narrative review for generating an extensive list of perceptions and reasons regarding e-cigarettes. The results were not limited to cross-sectional surveys with probability samples, or close-ended response options, but also include qualitative work. A deductive thematic analysis was performed to identify themes that appeared salient to the constructs: perceptions regarding e-cigarettes and reasons for e-cigarette use. The main constructs “Perceptions” and “Reasons” were used to categorize the major relevant findings in supplementary Table S3. The themes (for example, for reasons for e-cigarette use: “expected benefits” and “social environment”) were used to extract major relevant findings (supplementary Table S3). Kim A.G.J. Romijnders and Reinskje Talhout formulated sub-themes after extracting relevant findings for perceptions about e-cigarettes and reasons for e-cigarette use. The sub-themes were salient to the themes, for example, for the theme “perceived safety of use”, the sub-theme “perceived safety of ingredients” emerged as pertinent from the major relevant findings. Kim A.G.J. Romijnders coded the major relevant findings found in supplementary Table S3 according to the themes and sub-themes (e.g., for theme “expected benefits” the sub-themes “weight control” and “helps with concentration” were applied). Kim A.G.J. Romijnders and Reinskje Talhout agreed upon the themes and sub-themes before the coding of the major relevant findings took place. The coding led to an overview of perceptions of risks of e-cigarettes, perceived benefits of e-cigarettes, and reasons for e-cigarette use. To ensure the reliability of the meaning of themes and sub-themes during coding of articles, triangulation was used.

After coding, results were stratified by type of user and age. Adults were categorized as eighteen years or older, and youth were categorized as younger than eighteen years old. For each type of user, there was variability in reporting. For example, some studies report current use of e-cigarettes among current cigarette smokers without categorizing them as dual users, whereas other studies reported the current use of e-cigarettes with simultaneous current tobacco cigarette use as dual use [32,33]. Therefore, measures for type of user were recorded for each included study as defined by the respective authors (Table S3). This review categorized perceptions and reasons regarding e-cigarettes using the classification of users as stated in the original study. E-cigarette users are users of e-cigarettes without differentiating for frequency of use, co-current use or past use of cigarettes. Similarly, no distinction was made among cigarette smokers concerning frequency, lifetime use, co-current or past use of other tobacco products or e-cigarettes. If an included study mentioned perceptions or reasons regarding e-cigarettes among dual users, this review categorized these perceptions and reasons among dual users. Similarly, non-users were classified as not using e-cigarettes or cigarettes. No distinction was made between former users or users that had never smoked. Summarizing, type of users were categorized according to their original type of user classification without an attempt to synthesize type of user across studies. 

## 3. Results

A total of 65 studies from 72 articles met the eligibility criteria (see Figure 1) [25,32,33,34,35,36,37,38,39,40,41,42,43,44,45,46,47,48,49,50,51,52,53,54,55,56,57,58,59,60,61,62,63,64,65,66,67,68,69,70,71,72,73,74,75,76,77,78,79,80,81,82,83,84,85,86,87,88,89,90,91,92,93,94,95,96,97,98,99,100,101,102]. Articles report perceptions and reasons regarding e-cigarettes in 49 studies among e-cigarette users, 39 studies among cigarette smokers, 11 studies among dual users, and 19 studies among non-users, which are listed in an overview. The sample size ranged from 14 to 25,029 respondents. Most studies were conducted in the U.S. (*n =* 49), but studies were also conducted in the UK (*n =* 11), New Zealand (*n =* 6), Canada (*n =* 4), France (*n =* 2), Switzerland (*n* = 3), Australia (*n* = 2), and Belgium (*n* = 1) (see Table S2 for a full overview). Fifty-five articles reported data on adults, and seventeen on youth. Thirty-four studies had a cross-sectional design, seventeen had a qualitative design, three had a mixed methods approach, six were longitudinal, and twelve were cohort studies (see Table S3). Due to a variety of research designs, sample size, and changes over time, this paper is not a synthesis of most cited, most important, or most expressed perceptions and reasons by participants [23]. This section provides an overview of risk perceptions, perceived benefits, and reasons for e-cigarette use.

### 3.1. Risk Perceptions Related to E-Cigarettes

Perceived risks pertained to risks for individual e-cigarette users (e.g., unsafe components of e-liquids), and risks for the social environment of these users (e.g., risks for bystanders and the risk for an unborn child if used during pregnancy) [25,32,34,36,37,39,40,41,45,47,49,50,52,54,56,57,59,60,61,63,65,66,68,70,72,73,77,78,79,81,83,85,86,88,89,93,94,95,97,98,101,102]. Table 1 summarizes the different risk perception themes and sub-themes identified. This section reports perceptions mentioned by user groups. First, studies suggest that, compared to cigarettes, e-cigarettes were perceived by all user groups as being healthier, safer, and less addictive, as well as being safer for one’s social environment, and safer to use during pregnancy than cigarettes [32,37,39,40,41,43,44,47,48,49,57,59,61,63,65,68,73,76,81,85,88,89,94,95,102]. Second, studies performed in earlier years showed that e-cigarettes were perceived as being overall less harmful than cigarettes, while in later years this reduced harm perception changed [25,32,34,36,37,39,40,41,45,47,49,50,52,54,56,57,59,60,61,63,65,66,68,70,72,73,77,78,79,81,83,85,86,88,89,93,94,95,97,98,101,102]. In more recent studies e-cigarettes were perceived as equally or more harmful than cigarettes among adult cigarette smokers [25,36,37,47,52,59,66], non-users [54,60,72,73,85,93,94], as well as youth cigarette smokers [25,36,37,52,56,59,66], and non-users [25,47,52]. Third, specific flavors (candy and fruit flavors) were considered less harmful than other (tobacco) flavors among adult [49] and youth [56] e-cigarette users, adult [78] and youth [25,56] cigarette smokers, and non-users [56]. Summarizing, different themes and subthemes with regard to perceived risks for the individual e-cigarette user and risks for their social environment were specified. Flavors influence the risk perception of e-cigarettes among both adults and youth, and current data show that the risk perception of e-cigarettes increased compared to previous years. 

### 3.2. Perceived Benefits of E-Cigarettes

Perceived benefits of e-cigarettes mentioned in the literature are summarized in Table 2 and include (expected and actual) positive experiences (such as taste), social acceptance, avoidance of smoking restrictions, a cool and fashionable product, an effective smoking aid, and the safety for bystanders. In this section, perceived benefits of e-cigarettes for the user groups are shown. First, adult e-cigarette users [40,49,57,58,73,81,88,90,102] and adult cigarette smokers [37,39,42,50,63,68,70,78,88,89,99] noted health benefits and positive experiences of e-cigarette use. Dual users and non-users did not identify health benefits or positive experiences, although they did note some benefits for reducing cravings and safety for the e-cigarette user compared to cigarette smokers. Second, adult e-cigarette users [40,41,49,57,81,85], dual users [81], and non-users [58,85,94,95] also saw benefits for bystanders of e-cigarette users. Third, youth noted only a few perceived benefits of e-cigarette use for individual use. They perceived e-cigarettes as safe to use for e-cigarette users and fashionable (youth e-cigarette users [37,47,59], youth cigarette smokers [37,59], and youth non-users [47,55]). Summarizing, individual user benefits revolved around convenience and attractiveness of the product, health benefits, positive experiences, safety, smoking cessation benefits, and social acceptability. Perceived benefits for the social environment of the user were mentioned by adult user groups (safety for bystanders and the environment). 

### 3.3. Reasons for E-Cigarette Use 

This section reports reasons for use among e-cigarette users, cigarette smokers, dual users, and non-users. Non-users were asked about possible reasons for them to personally initiate e-cigarette use. Reasons for explaining the appeal of e-cigarettes go beyond smoking cessation (Table 3; [32,33,35,37,39,40,41,42,43,44,46,48,49,50,51,53,54,57,62,64,65,66,67,69,70,71,74,75,76,79,81,82,83,84,85,86,90,91,98,102]). Other reasons include expected benefits (enjoyable taste and a variety of flavors), experienced benefits (reduces stress and enables control of weight gain), avoidance of smoking restrictions by dual use of tobacco products and e-cigarettes, convenience of the product, curiosity, and influences from the social environment (e.g., recommended by friends). Smoking cessation was the most often reported reason for initiation of e-cigarette use among adult e-cigarette users [33,35,40,41,42,44,48,49,51,53,54,57,62,64,65,66,69,75,76,79,81,82,85,86,90,91,102], cigarette smokers [39,42,43,46,50,69,70,71,76,83,84,98], dual users [32,33,67,81,82], and non-users [76]. In addition, other expected benefits were reported by adult [35,40,41,48,49,53,65,76,79,81,90] and youth [44,59,75] e-cigarette users, adult [46,76,84] and youth [59] cigarette smokers, dual users [32,48], and adult non-users [76] (see Table 3). In addition to expected benefits, adult [33,35,40,41,48,53,65,74,76,79,81,85,90,91] and youth [44,59,75] e-cigarette users and dual users [32,33,48,81] reported additional experienced benefits such as health benefits and finding a new hobby. In summary, reasons for e-cigarette use go beyond smoking cessation. While smoking cessation is the reason most often reported in large-scale population surveys, most other reported reasons revolved around the health benefits of e-cigarette use compared to smoking.

## 4. Discussion

This review provides a comprehensive overview of risk perceptions, perceived benefits, and reasons for use of e-cigarettes, as reported in Table 1, Table 2 and Table 3.

### 4.1. Perceptions and Reasons Among Users and Non-Users

Current data showed a variety of perceptions about e-cigarettes and reasons for e-cigarette use reported by e-cigarette users, cigarette smokers, dual users, and non-users. For example, e-cigarettes were perceived as being less harmful by e-cigarette users. This perception of reduced harm could lead to use or, vice versa, by initiating e-cigarette use, the perception of harm may decrease. However, research showed that the perceived harm of e-cigarettes as compared to tobacco cigarettes has increased among all types of users over the years [3], and e-cigarettes are currently perceived as equally or more harmful than cigarettes. With regard to available flavors, which were shown to influence risk perceptions, fruit or candy flavored e-liquids were perceived as less risky compared to tobacco flavored e-liquids. E-cigarette users and cigarette smokers perceived benefits of e-cigarettes. In addition, adult e-cigarette users, dual users, and non-users noted advantages for the social environment when switching from cigarette smoking to e-cigarette use. Youth highlighted the trendiness of e-cigarettes as a perceived benefit, and perceived less health benefits than adults. The overview in this paper shows several positive perceptions and reasons which influence the initiation of e-cigarette use.

Based on these findings, tailored communication on risks and benefits of e-cigarette use could increase awareness about risks and benefits of e-cigarette use among user groups. For example, targeted risk communication on risks of e-cigarette use for non-users, and benefits of e-cigarette use compared to smoking for cigarette smokers would increase factual knowledge about risks of e-cigarette use among these user groups. If the latter were to perceive e-cigarettes as less harmful, they may be more inclined to switch to e-cigarettes. Furthermore, if non-users were not to perceive fruit- and candy-flavored e-liquids as harmless, they might be less inclined to initiate e-cigarette use. Summarizing, risks and benefits could be communicated to increase knowledge about e-cigarette use among user groups.

E-cigarette users expected (before initiation) and experienced (after continuation of use) benefits from e-cigarette use. In addition, this paper noted that reasons for initiation of e-cigarette use evolved to reasons for continuation of e-cigarette use [14]. When positive outcome expectancies (theme: expected benefits, see Table 3) [32,33,40,41,42,43,46,47,49,50,53,75,100,103] were realized by positive experiences when initiating e-cigarette use (theme: experienced benefits, see Table 3) [32,33,40,41,49,53,75,84], people may continue using e-cigarettes. For example, all e-cigarette users expected health benefits from e-cigarette use compared to cigarettes [32,33,40,41,42,43,46,49,53,75,100]. If health improvements are indeed experienced, this may lead to continued use of e-cigarettes and possibly quitting cigarette use [32,33,40,49,53,75] (see Table 3).

Cigarette smokers and non-users also mentioned expected benefits from e-cigarette use. However, not all cigarette smokers continue with e-cigarette use after initiation or initiate e-cigarette use. In some cases, the expected benefits of e-cigarettes for cigarette smokers—the ability to mimic smoking behavior—did not result in the expected experience. Cigarette smokers who tried e-cigarettes often expressed the inability to mimic smoking behavior with an e-cigarette (e.g., as a result of taste, the weight of the device, not being able to hold the device in the same way as a tobacco cigarette) [33,41,42,43,49]. The experience of e-cigarettes did not live up to the outcome expectations of cigarette smokers. Consequently, managing outcome expectations (by assisting with device specifications choices or e-liquid flavors) in behavior change strategies for cigarette smokers may prevent dual use of e-cigarettes and cigarettes. Managing outcome expectations could also be used to prevent initiation among non-users, by focusing on expected disadvantages of use and negative experiences (such as stressing that it is not cool or fashionable to use e-cigarettes).

### 4.2. Applications

Perceptions and reasons regarding e-cigarette use provide additional input for public health education, behavioral change programs, and regulation. Regulation, such as warning labels on tobacco products, is used to target misperceptions regarding tobacco products on a population level. Public health education can use the overview, presented in this study of perceptions on risks and benefits, to highlight factual risks and benefits of e-cigarette use in tailored communication. For example, tailored risk communication on the reduced harmfulness of e-cigarettes compared to cigarettes may reduce misperceptions among cigarette smokers initiating e-cigarette use for smoking cessation purposes. With risk communication tailored to specific personal needs and personal outcome expectancies, behavior change experts are able to target these personal misperceptions, and confirm factual risk perceptions and perceived benefits. 

Policy makers can also use this overview for product regulation measures. For example, available e-liquid flavors play an important role in the initiation of e-cigarette use for both cigarette smokers looking for an alternative for cigarettes and for curious non-users [9]. From a public health point of view, it is not desirable for non-users to be attracted by flavors in e-liquids, with the chance of initiating e-cigarette use. Future research should therefore focus on differences and overlap in specific flavor preferences among cigarette smokers and non-users to facilitate switching from cigarettes to e-cigarettes and discourage initiation of e-cigarette use among non-users [22,25].

### 4.3. Future Research

Heterogeneity in the reporting of types of users made it difficult to classify types of users. For future research, it is therefore of vital importance to formulate standard definitions for ever, current, and dual use of e-cigarettes to assess population effects of e-cigarette use. In defining e-cigarette use, it is important to distinguish between experimental and daily use. For example, asking about e-cigarette use during the previous 30 days does not distinguish between experimental and daily use.

This review noticed the lack of reporting on perceptions towards e-cigarettes and reasons for use among adult dual users and non-users, and youth non-users and dual users. Future research needs to identify the rates of dual use among youth. Overall, only perceptions of harm were assessed in extensive cross-sectional, cohort, and longitudinal studies compared to other risk perceptions, and less regarding perceptions in general.

E-cigarette use is a complex behavior, and response options in questionnaires assessing perceptions and reasons in general may not be representative for all users, cigarette smokers, dual users, and non-users. Our overview, in addition to the work of Gibson et al. [26] and Pearson et al. [24], validated measures such as the Fagerström test for nicotine dependence [104] and the International Tobacco Control measures [105], and adds insight into developing and validating items for measuring e-cigarette use, risk perceptions of e-cigarettes, perceived benefits of e-cigarettes, and reasons for e-cigarette use. Summarizing, validated measures provide insight into e-cigarette use to develop tailored information based on the needs of e-cigarette users, cigarette smokers, dual users, and non-users. 

### 4.4. Limitations

Heterogeneity between the different papers in statistical methods and reporting makes it difficult to generalize findings across countries and study samples. Therefore, the results do not display analyses across countries. For this reason, the current paper was unable to display changes in risk perception over time. Due to the variability in reporting type of users and frequency of use, users were classified according to the classification of original articles. Consequently, this overview was unable to differentiate between former and never e-cigarette users, or to clearly differentiate between cigarette smokers and dual users, as not all cigarette smokers currently using e-cigarettes were classified in original studies as dual users of tobacco and e-cigarettes. In addition to a variety in study designs, more studies were found reporting on adult perceptions and reasons than youth, and cigarette smokers than non-users. This means that some perceptions and reasons regarding e-cigarettes could have been missed among the understudied user groups.

## 5. Conclusions

This study is an exploratory narrative review into perceptions and reasons regarding e-cigarette use. Different perceptions of risks and benefits, and reasons for e-cigarette use were summarized for different types of users in themes and sub-themes, such as convenience, social environment, and disadvantages. Adults’ perceptions and reasons for e-cigarette use are often related to smoking cessation, while youth like the novelty of the product. Tailored information about e-cigarettes for the different user groups is necessary to correct misperceptions about e-cigarettes and highlight the risks and benefits of e-cigarette use.

For public health professionals, behavior change experts, and regulatory science, our overview of risk and benefit perceptions of e-cigarettes, and reasons for e-cigarette use provides insight into the initiation of e-cigarette use. 

## Figures and Tables

**Table 1:** Risk perceptions about e-cigarettes reported by individual studies, clustered by type of user.

Themes and Sub-Themes of Risk Percpetions	E-Cigarette Users ^a^		Smokers ^b^		Dual Users ^c^		Non-Users ^d^	
	Adults	Youth	Adults	Youth	Adults	Youth	Adults	Youth
**Risk Perceptions for the User**								
**Harmfulness**	[40,49,54,57,59,73,79,85,86,94]	[25,36,37,47,52,59,66]	[34,39,41,45,50,54,59,60,70,72,77,83,85,88,93,97,98,101]	[25,36,37,52,56,59,66]	[32]		[54,60,72,73,85]	[25,47,52]
Harmfulness of flavors	[49]	[56]	[78]	[25,56]				[56]
Secondary harm as a gateway drug							[94]	
**Health risks**	[49,65]	[37,47]	[65]					
Reduced athletic performance	[49]							
Trouble breathing/Coughing	[49]	[47]						
Cancer	[49]	[47]						
Hearth attack	[49]							
Dental health issues	[49]							
**Safety of use**	[37,40,57,59,102]	[37,59]	[63,68,88,89]	[37,59]	[63]			
Lack of safety of ingredients liquids		[59]		[59]				
**Risk Perception for the Social Environment of an user**								
**Harmful for bystanders**	[40,57,81,85]						[85]	
**Safety of use during pregnancy**							[61,73]	

Note: ^a^ “E-cigarette users” are users of e-cigarettes as defined in the original study. For example, Bold, Kong, Cavallo, Camenga and Krishnan-Sarin [44] included only ever users of e-cigarettes in their study, without differentiating for frequency of use, co-current use or past use of cigarettes. ^b^ “Smokers” are those who smoke cigarettes as defined in the original studies. For example, Biener, Song, Sutfin, Spangler and Wolfson [43] defined cigarette smokers as those who had smoked at least 100 cigarettes in their lifetime and smoked every day or some days. No distinction is made among cigarette smokers concerning frequency, lifetime use, co-current or past use of other tobacco products or e-cigarettes. ^c^ “Dual users” are those who use e-cigarettes and cigarettes simultaneously as defined in the original studies. For example, Cheney, Gowin and Wann [48] defined dual users as current use of both e-cigarettes and cigarettes within the past week. If an included study mentioned perceptions or reasons regarding e-cigarettes among dual users, this review categorized these perceptions and reasons among dual users. ^d^ “Non-users” are those who did not use e-cigarettes or cigarettes at the time of included study as defined in the original study. For example, Patel, Davis, Cox, Bradfield, King, Shafer, Caraballo and Bunnell [76] defined non-users as those who report “not at all” to the question whether they had smoked cigarettes or used e-cigarettes. Non-users were classified as not using e-cigarettes or cigarettes, and no difference was made between former users and never users.

**Table 2:** Perceived benefits of e-cigarettes reported by individual studies, categorized by type of user.

Perceived Benefits for Users								
Themes and Sub-Themes of Perceived Benefits	E-Cigarette Users ^a^		Smokers ^b^		Dual Users ^c^		Non-Users ^d^	
	Adults	Youth	Adults	Youth	Adults	Youth	Adults	Youth
**Addictiveness**	Perceived as less addictive [73,81,94]	[47,59]	Perceived as equally addictive [39,41]	[59]	Perceived as less addictive [32,81]		Perceived as equally addictive [73,94]	[47]
**Avoidance of smoking restrictions**	[49]	[37]	[50,63,68,88,89]	[37]	[63]		[73]	
**A cool and fashionable product**	[73]	[37,47]		[37]			[73]	[47,55]
**Health benefits**	[90]							
Healthier than cigarettes		[37]	[42]	[37]				
Improved breathing			[63]		[63]			
Improved general well-being			[63,78]		[63]			
Decreased coughing			[63]		[63]			
Less likely to cause cancer			[78]					
**Lower costs compared to cigarettes**			[50]					
**Positive experiences**								
Mimics smoking routine		[37]	[37]	[37]				[55]
Enjoyable taste	[81]				[81]		[73]	
Throat hit	[81]				[81]			
Weight control	[81]				[81]			
Increases concentration		[47]						
**Safety of use**	[40,57,102]	[37,59]	[63,68,88,89]	[37,59]	[63]			
Safety of ingredients liquids	[73]	[59]		[59]				[47,55]
**Smoking cessation purposes**								
Nicotine replacement therapy	[49,58,73,88]		[39,50,70,99]		[32,89]		[58,61,73,88,94]	
Cut back on cigarettes			[50]					
Deal with cravings					[89]		[58]	
**Social acceptability**	[41,49,81]				[81]		[58,94,95]	
**Perceived Benefits for the Social Environment of an User**								
**Safer for bystanders**	[40,57,81,85]				[81]		[85]	
**Safer for the environment (less pollution)**	[57]							

Note: ^a^ “E-cigarette users” are users of e-cigarettes as defined in the original study. For example, Bold, Kong, Cavallo, Camenga and Krishnan-Sarin [44] included only ever users of e-cigarettes in their study, without differentiating for frequency of use, co-current use or past use of cigarettes. ^b^ “Smokers” are those who smoke cigarettes as defined in the original studies. For example, Biener, Song, Sutfin, Spangler and Wolfson [43] defined cigarette smokers as those who had at least smoked 100 cigarettes in their lifetime and smoked every day or some days. No distinction is made among cigarette smokers concerning frequency, lifetime use, co-current or past use of other tobacco products or e-cigarettes. ^c^ “Dual users” are those who use e-cigarettes and cigarettes simultaneously as defined in the original studies. For example, Cheney, Gowin and Wann [48] defined dual users as current users of both e-cigarettes and cigarettes within the past week. If an included study mentioned perceptions or reasons regarding e-cigarettes among dual users, this review categorized these perceptions and reasons among dual users. ^d^ “Non-users” are those who did not use e-cigarettes or cigarettes at the time of included study as defined in the original study. For example, Patel, Davis, Cox, Bradfield, King, Shafer, Caraballo and Bunnell [76] defined non-users as those who report “not at all” to the question of whether they had smoked cigarettes or used e-cigarettes. Non-users were classified as not using e-cigarettes or cigarettes, and no difference was made between former users or never users.

**Table 3:** Reasons for e-cigarette use as reported by individual studies by type of user.

Themes and Sub-Themes of Reasons for E-Cigarette Use	E-Cigarette Users ^a^		Smokers ^b^		Dual Users ^c^		Non-Users ^d^	
	Adults	Youth	Adults	Youth	Adults	Youth	Adults	Youth
**Expected benefits**								
Enjoyable taste	[40,53,65,81]		[46]					
Expected health benefits								
Healthier than cigarettes	[33,40,41,49,53,57,81]	[44,75,80]			[32,33]			
Improved breathing	[91]	[37,47]		[37]				
Increased concentration					[32]			[47]
Satisfy nicotine need	[38,90]				[32]			
Availability of variety of flavors	[35,40,41,48,49,65,76,79]	[44,59]	[76,84]	[59]	[48]		[76]	
Weight control	[41,81]	[75]						
**Experienced benefits**								
Avoidance of smoking restrictions by dual use of tobacco products and e-cigarettes	[35,65]	[37,92,100]	[42,43,50,69,76,83,84]	[37]	[82]		[76]	[95]
Possibility to alter technical specifications	[40,74,90]							
Weight control	[41,81]							
Mimics smoking routine	[33,40,49,54,79,91]	[37]		[37]	[33]			
Experienced health benefits	[33,40,48,49,53,81]	[37,75]		[37]	[32,33]			
Regain a sense of smell and taste	[40,53]							
Improved breathing								
Decreased coughing								
Improved dental health								
Increased athletic performance		[47,75]						
Increased alertness								
New hobby (more friends)	[33,48,91]				[33,48]			
Aid to concentration					[32]			[47]
Pleasure of product use	[33,40,53,85,91]				[33]			
Reduces stress	[48,81]		[84]		[81]			
Taste of flavors	[35,40,41,48,65,76,79,81]	[44,59]	[76,84]	[59]	[32,33]		[76]	
Throat hit	[40,81]		[84]		[81]			
**Convenience of product**	[91]							
Easily accessible	[40,48,71,91]	[37,80]	[71]	[37]				
Lower costs compared to cigarettes	[33,38,40,41,69,74,79,85,87,96]	[37,75,100]		[37]	[32,33]			
Discreet in use (no lingering smell, able to hide use)		[44]						
Practical in use (no lighter, no ashtray, one puff, and able to store the device)	[40,48,71,76,91]		[71,76]		[48,76]		[76]	
**Curiosity**								
A cool product	[35,69,76,79,81,87,96,98]	[44,47,66,100]	[39,71,76,98]	[66]	[81]		[39,76]	
A fashionable product		[37]		[37]				
Novelty (curious about novel product)	[35,53,65,69,70,76,79,81,85]	[44,62,64,66,92,100]	[39,43,69,71,76,84,98]				[76]	
**Smoking cessation purposes**								
Alternative for smoking cigarettes	[38,57,86]		[69]					
Avoidance of withdrawal of nicotine	[38,53]							
Cut back cigarettes	[33,42,79,81,87,96]	[75,92,100]	[50,83]		[32,33,81]			
Use as smoking cessation aid	[33,35,38,40,41,42,44,51,53,54,62,64,65,66,69,75,76,79,81,85,87,90,91,102]	[44,51,62,64,66,75,92]	[39,42,43,46,50,70,76,83,84,98]	[100]	[32,33,67]		[76]	
Deal with cravings	[40,54,76,79,82,85,96]		[71,76]		[32,33,82]		[76]	
**Social environment**								
Fitting in								[47]
Pressure of social environment			[41]					[47]
Recommended by friends or family	[96]	[92,100]	[69]					
Role models use e-cigarettes	[41,49]	[92]						

Note: ^a^ “E-cigarette users” are users of e-cigarettes as defined in the original study. For example, Bold, Kong, Cavallo, Camenga and Krishnan-Sarin [44] included only ever users of e-cigarettes in their study, without differentiating for frequency of use, co-current use or past use of cigarettes. ^b^ “Smokers” are those who smoke cigarettes as defined in the original studies. For example, Biener, Song, Sutfin, Spangler and Wolfson [43] defined cigarette smokers as those who had at least smoked 100 cigarettes in their lifetime and smoked every day or some days. No distinction is made among cigarette smokers concerning frequency, lifetime use, co-current or past use of other tobacco products or e-cigarettes. ^c^ “Dual users” are those who use e-cigarettes and cigarettes simultaneously as defined in the original studies. For example, Cheney, Gowin and Wann [48] defined dual users as current use of both e-cigarettes and cigarettes within the past week. If an included study mentioned perceptions or reasons regarding e-cigarettes among dual users, this review categorized these perceptions and reasons among dual users. ^d^ “Non-users” are those who did not use e-cigarettes or cigarettes at the time of included study as defined in the original study. For example, Patel, Davis, Cox, Bradfield, King, Shafer, Caraballo and Bunnell [76] defined non-users as those who report “not at all” to the question of whether they had smoked cigarettes or used e-cigarettes. Non-users were classified as not using e-cigarettes or cigarettes, and no difference was made between former users and never users.

## Supplementary Materials

The following are available online at http://www.mdpi.com/1660-4601/15/6/1190/s1, Table S1: Search Strategy for OvidMedline^®^_Original search, Table S2: Decision Tree of Exclusion Criteria, Table S3: Major Relevant Findings of Included Articles.
