# Supplementary material for: Perceptions and Reasons Regarding E-Cigarette Use among Users and Non-Users: A Narrative Literature Review
Source: Int J Environ Res Public Health. 2018 Jun 6;15(6):1190. doi: 10.3390/ijerph15061190 (PMC6025300; doi:10.3390/ijerph15061190)
Supplement: Supplementary file 1 [file ijerph-15-01190-s001.zip › ijerph-300843/Table S1.docx]

Table S1. Search Strategy for OvidMedline®_Original search.

| Data Source | Provider/Interface | Ovid |
| --- | --- | --- |
|  | Database | Medline® |
|  | Date searched | 09-02-2016 |
|  | Database update | 09-02-2017 |
|  | Search developer(s) | Wim ten Have & Kim Romijnders |
| Limiters & other information | English only? (default is "y") | y |
|  | Date restrictions | 1950–present |
| Note: an asterisk is used as a wildcard symbol to broad the search by including words that start with the letters before the asterisk. | | |
| **Search Strategy** | | |
|  | 1 | (electr* adj (cigar* or nicotine)).tw. (803) |
|  | 2 | ((electronic or electrically) adj3 (cigar* or nicotine)).tw. (852) |
|  | 3 | (e-cig* or ecig* or e-cigarette* or ecigarette* or e-nicotine* or enicotine*).tw. (846) |
|  | 4 | electronic cigarettes/(439) |
|  | 5 | 1 or 2 or 3 or 4 (1316) |
|  | 6 | (initiation or motivation* or motives or reason* or attitude* or belief* or expectan* or understanding or knowledge or reactions or awareness or perception* or opinion or views or debate* or discussion* or image or popularity or desirab* or demand).ti. (409599) |
|  | 7 | ((frequency adj5 ("use" or usage)) or (real-world adj5 ("use" or usage))).tw. (12469) |
|  | 8 | (("use" or usage) and (frequency or prevalence or among or change* or emerg* or factors or associations or trends or traditional or conventional or locations)).ti. (31844) |
|  | 9 | motivation/or perception/or awareness/or attitude/or attitude to health/or public opinion/or consensus/or focus groups/or social desirability/or consumer behavior/ (252874) |
|  | 10 | (internet or social media or facebook or twitter or youtube).ti. or internet/ or social media/(59745) |
|  | 11 | 5 and (6 or 7 or 8 or 9 or 10) (300) |
|  | 12 | (vaping or behavio?r*).ti. or (smoking sensation or smoking characteristics or sensation seeking).tw. or sensation/ or smoking/px or electronic cigarettes/px (247982) |
|  | 13 | 5 and 12 (135) |
|  | 14 | harm reduction.ti. or harm reduction.ot. or harm reduction/ (2185) |
|  | 15 | 5 and 14 (68) |
|  | 16 | (relative harm or product harm or cessation).ti. or (relative harm or product harm or cessation).ot. (11395) |
|  | 17 | smoking cessation/ or "tobacco use cessation"/ or health behavior/ or risk reduction behavior/ or treatment outcome/ (768920) |
|  | 18 | (effect or effects or efficacy or effectiv* or benefit or evidence* or consequences or impact or success* or public support or comparison or compared or control policies or strateg* or cochrane or foundation).ti. (2501872) |
|  | 19 | 5 and (16 or 17) and 18 (58) |
|  | 20 | (marketing or advertis* or commercials or communicating or communicat*).ti. (60382) |
|  | 21 | marketing/ or commerce/ or communication/ or exp advertising as topic/ or advertisements.pt. (102541) |
|  | 22 | 5 and (20 or 21) (131) |
|  | 23 | (policy or policies or strateg* or public health or legislat* or regulat* or guideline* or government* or ban or bans or taxes or tax or taxation*).ti. (635686) |
|  | 24 | health policy/ or public policy/ or public health/ or social control policies/ or social control, formal/ or government regulation/ or legislation, medical/ or smoke-free policy/ or air pollution, indoor/lj or workplace/lj or restaurants/lj or smoking/lj or taxes/ or health promotion/ (235485) |
|  | 25 | (guideline or legisation).pt. (15644) |
|  | 26 | 5 and (23 or 24 or 25) (274) |
|  | 27 | (effect or effects or efficacy or effectiv* or benefit or evidence* or consequences or impact or success* or public support or comparison).ti. (2373199) |
|  | 28 | 26 and 27 (24) |
|  | 29 | (tobacco product*.ti. or tobacco products/) and (harm*.ti. or harm*.ot. or harm reduction/) (72) |
|  | 30 | 11 or 13 or 15 or 19 or 22 or 28 or 29 (605) |
|  | 31 | (english or dutch).lg. (20815326) |
|  | 32 | 30 and 31 (591) |
|  | 33 | (letter or comment or news).pt. (1342230) |
|  | 34 | 32 not 33 (505) |
|  | 35 | remove duplicates from 34 (498) |
