# Supplementary material for: Perceptions and Reasons Regarding E-Cigarette Use among Users and Non-Users: A Narrative Literature Review
Source: Int J Environ Res Public Health. 2018 Jun 6;15(6):1190. doi: 10.3390/ijerph15061190 (PMC6025300; doi:10.3390/ijerph15061190)
Supplement: Supplementary file 1 [file ijerph-15-01190-s001.zip › ijerph-300843/Table S2.docx]

# Table S2. Decision tree_Exclusion criteria. (These exclusion criteria are hierarchical in order, meaning if reasons #1 applies as a reason for exclusion, the other reasons do not have to be noted down.)

| **#1 not e-cigarette** | |
| --- | --- |
| Does the article describe a subject which is not the electronic cigarette (e-cigarette), vaping/vapor? often ‘’e-cigarette/vaping’’ etc. is not mentioned in the title? | Does the article describe tobacco, smoking or alternative tobacco products without mentioning the e-cigarette? |
| Does the article describe a subject which is | Does the article describe a subject which is |
| Yes? EXCLUDED based on exclusion criterion, #1. | |
| No? Move on to exclusion criterion #2. | |
| **#2 toxicology and vaping behavior** | |
| Does the article discuss the chemical composition of alternative tobacco products (e-cigarettes are thus clearly mention (otherwise excluded at #1)? | - psychopharmacological properties, - chemical characteristics, - also testing for toxicity or safety, toxicological properties - pharmacological attributes of nicotine, - components of e-liquids (Gas Chromatography-Mass Spectrometry analyses), - mention aerosol, air quality, - Topography, - Effectiveness (not in relation to harm reduction) of nicotine delivery - Safety/risks of an e-cigarette (use), - or ingredients, - burden of disease or - innovations regarding e-cigarettes? - health as an outcome, - nicotine health risks, - lung disease, - cancer, - mental illness, - blood levels - nicotine intake, - cravings (not as a subjective reasons given for e-cigarette use, but tested (e.g., experimental setting) - comorbidity/relation with disease/mental - dependency (i.e., drug/alcohol use in combination with e-cigarette use) |
| Does the article describe the physiological response to e-cigarette use? |  |
| Does the article describe toxicological effects of e-cigarette use? |  |
| Does the article describe the use of non-human subjects? |  |
| Yes? EXCLUDED based on exclusion criterion #2. | |
| No? Move on to exclusion criterion #3. | |
| **#3 opinion article** | |
| Does the title or abstract revolve around or phrased in a way an opinion or stands with regard to the e-cigarette? | For example, feelings about the e-cigarette debate, or opinions about the e-cigarette or titles that suggests an opinion towards the e-cigarette, or analyzing debates/opinions? Usually, the title is framed as a question (e.g., ‘’E-cigarettes: a nicotine gateway?’’) |
| Yes? EXCLUDED based on exclusion criterion #3 | |
| No? Move on to exclusion criterion #4 | |
| **#4 market or marketing** | |
| Does the article describe the market of alternative tobacco products (specifically mentioning e-cigarettes)? | Not related to participants describing awareness of e-cigarettes as a result of marketing strategies |
| Does the article describe marketing/advertising of alternative tobacco products (specifically mentioning e-cigarettes)? |  |
| Examples | - Market trends, - Sale points of e-cigarettes, - Retail, - Types of e-cigarettes on the market, - The increase in sales of e-cigarettes - The role of advertising on use |
| Yes? EXCLUDED based on exclusion criterion #4. | |
| No? move on to exclusion criterion #5. | |
| **#5 harm reduction** | |
| Is the article about harm reduction/smoking cessation? | EXCEPTION: But Not Perceptions of Study Participants Regarding Perceptions of Harm or Perceived Effectiveness as Smoking Cessation. |
|  | Other than participants describing the use of an e-cigarette as a form of harm perceptions or as a reason why the use the e-cigarette? |
| Does the article discuss smoking cessation, with the e-cigarette as a tool | - Measured effectiveness as smoking cessation aid - Evaluated the intention to quit smoking using e-cigarettes |
| Does the article discuss the public health debate surround the e-cigarette? | |
| Yes? EXCLUDED based on exclusion criterion 5# | |
| No? move on to exclusion criterion #6 | |
| **#6 e-cigarette regulation** | |
| Does the article revolve around policy work/regulation/tobacco control/policy recommendations? | - Asking for policy change, - Describes policy change with regard to the e-cigarette, - The e-cigarette as a form of harm reduction policy (other than users describing perceptions of harm), - The e-cigarette in relation to tobacco control? |
| Yes? EXCLUDED based on exclusion criterion #6 | |
| No? move on to exclusion criterion #7 | |
| **#7 subjective reports** | |
| Does the article describe the use of the e-cigarette by as rehabilitation? | - The use of e-cigarettes in out-patient centers, - Psychiatric clinics etc. - How to use the e-cigarette? - Opinion on use other than users opinions themselves - No subjective reports of perceptions or reasons provided - Only description of awareness and patterns of use. - Alternative use for other drugs, such as marijuana. |
| Does the article describe the use of e-cigarettes in institutionalized participants (not stop smoking services) |  |
| Does the article describe patterns of awareness? |  |
| Does the article describe patterns of use? |  |
| Yes? EXCLUDED based on exclusion criterion #7 | |
| No? move on to exclusion criterion #8 | |
| **#8 gateway effect** | |
| Does the article describe the uptake of e-cigarettes or e-cigarettes as a gateway to other tobacco/drug products by individuals? | EXCEPTION: Not the perception of participants that e-cigarettes are a gateway drug |
| Does the article describe the risk factors leading to uptake of e-cigarettes without describing subjective reports of perceptions and reasons? | EXCEPTION: Not if risk factors are the perceptions & reasons provided by subjective reports of participants |
| Yes? EXCLUDED based on exclusion criterion **#8** | |
| No? move on to exclusion criterion #9 | |
| **#9 not an article** | |
| Is it not an article, but newspaper dissertations, narratives, commentaries, gray literature, and editorials were excluded etc.? |  |
| **Yes, fill in ‘’NO’’, and as** exclusion criterion **#9** | |
| No? move on to exclusion criterion #10 | |
| No? Fill in ‘’MAYBE, appropriate for inclusion’’  (End of exclusion criteria screening, continue for full text) | |
| **#10 conflict of interest** | |
| Is there a conflict of interest? | - Financial relationship questionable - Related to the tobacco industry |
| **#11 age restriction apply** | |
| Does the article include samples with unclear age restrictions? | - The article mentions age restriction for grades, however, not specific for age. If the grade falls well within the age restriction of <18, that age restriction do not apply. If students can be 18 or 19. Age restriction apply |
| Does the article include a sample with overlap of adults and adolescents | - Age 15 to 19 years old - 17 to 21 years old - Etc. |
