# Supplementary material for: Perceptions and Reasons Regarding E-Cigarette Use among Users and Non-Users: A Narrative Literature Review
Source: Int J Environ Res Public Health. 2018 Jun 6;15(6):1190. doi: 10.3390/ijerph15061190 (PMC6025300; doi:10.3390/ijerph15061190)
Supplement: Supplementary file 1 [file ijerph-15-01190-s001.zip › ijerph-300843/Table S3.docx]

**Supplementary Table S3.** Main findings of included articles in alphabetical order.

|  | **First Author** | **N** | **Country** | **Brief study description** | **Mayor relevant findings** |
| --- | --- | --- | --- | --- | --- |
| [34] | Adkison, O'Connor, Bansal-Travers, Hyland, Borland, Yong, Cummings, McNeill, Thrasher, Hammond and Fong [34] | U.S. (n = 1520), UK (n = 1325), Canada (n = 1581), Australia (n = 1513): total n = 5939 | U.S., UK, Canada, Australia | International Tobacco Control (four-country) survey). Cross-sectional data from the U.S. (n = 1520), UK (n = 1325), Canada (n = 1581), Australia (n = 1513) total n = 5939.  Types of user as defined by authors: ‘Current smoker; smoked at least 100 cigarettes in their lifetime and at least 1 cigarette in the past 30 days. 18 years or older current (at least 100 cigarettes lifetime and 1 cigarette past 30 days) and former smokers.’ | PERCEPTIONS less harmful than traditional cigarettes: all: 70.3%; Canada: 63.9%; U.S.: 65.9%; UK: 82.2%; Australia: 71.0%. Perception of harm was higher in the US than UK (i.e. e-cigs are legal), and higher in Canada than Australia (i.e. e-cigs are banned). 79.8% using e-cigs because considered less harmful than traditional cigarettes; 75.4% used e-cigs to help reduce smoking; 85.1% using e-cigs to help quit smoking. Those who reported that e-cigs were less harmful than traditional cigarettes had nearly four times greater odds of trying e-cigs. (e-cigs users were more likely to have reduced their cigarettes per day between waves than non-users). |
| [35] | Amato, Boyle and Levy [35] | n = 9304 | U.S. | The 2014 Minnesota Adult Tobacco Survey; a cross-sectional sample of Minnesotan adults aged 18 years or older. Types of user as defined by authors: ‘Past users 0 days in past.  Infrequent users 1–5 days in past. Intermediate users 6–29 days in past. Daily users; Every day in past.’ | REASONS Goal oriented: To quit other tobacco products, to cut down, less harmful, to use them in places where other tobacco is not allowed, affordable. Non-goal oriented: curiosity, menthol flavor, other flavors. |
| [36] | Ambrose, Rostron, Johnson, Portnoy, Apelberg, Kaufman and Choiniere [36] | n = 24,658 | U.S. | National Youth Tobacco Survey (n = 24,658); cross-sectional.  Types of user as defined by authors: ‘Current smokers: individuals who reported smoking on. At least one of the past 30 days. Ever smokers: those who reported ever having tried cigarette smoking but not smoking within the past month. Dual use: any reported past 30 days use of e-cigarettes among current cigarette smokers. 11–18 years old; non-users, smokers and e-cigarette users current, (1 in past 30 days) ever and never cigarette smokers.’ | PERCEPTIONS 30.6% overall believed e-cigarettes to be less harmful than regular cigarettes, 25.0% of never smokers, 41.3% of ever smokers, and 54.2% of current smokers 64.2% perceived harmfulness cigarettes dose-dependent. |
| [37] | Anand, McGinty, O'Brien, Guenthner, Hahn and Martin [37] | n = 2769 | U.S. | Cross-sectional data from the U.S. (n = 2769) among youth.  Types of user as defined by authors: ‘Current use: past 30 day use. Ever use: lifetime use.’ 14–18 years old; e-cigarette users, smokers and other tobacco product users | PERCEPTIONS 60% minor health hazard. Healthier (7.5%), safer (6.9%), can be used anywhere (5.4%), do not have harmful chemicals (3.4%), mimic tobacco smoking (6.0%), trendier (3.5%), easier to get (2.0%), and more affordable (1.2%) safer (6.9%), no harmful chemicals (3.4%).  REASONS helped to smoking cessation (31.0%) 51% of e-cigarette users and 28% of none-cigarette users, accessibility (2.0%): (most accessible from friends (35.9%)), health benefits (7.5%), avoid smoking restriction (5.4%), mimic smoking (6.0%), trends (3.5. %), costs (1.2%). E-cigarettes most accessible from friends (35.9%) tobacco stores (23.5%), gas stations (17.2%), and family (13.2%). |
| [38] | [38] | n = 12 | Malaysia | Qualitative research (interviews) with 21–40 year old e-cigarette users.  Types of user as defined by authors: ‘Past and current users (have been using for <1 year, > 1 year, more than 2 year), of e-cigarettes). Past users are not specified.’ | REASONS Smoking cessation, cut back on smoking, alternative way of tobacco consumption, costs, health benefit, withdrawal symptoms, satisfaction |
| [39] | Bauhoff, Montero and Scharf [39] | n = 796 | U.S. | Cross-sectional survey using an online platform (n = 796).  Types of user as defined by authors: ‘Participants were never smokers (56%) or ever smokers (44%). 72% never tried e-cigarettes. 18 to 64 years old. Those who were aware of e-cigarettes were asked if they had “ever used” e-cigarettes and how often they used e-cigarettes in the last 30 days.’ | PERCEPTIONS believe that e-cigarettes can help smokers quit smoking (58%), less harmful than smoking (64%). not less addictive than smoking (27%). current smokers more likely that e-cigarettes could help smokers quit (64%). ever smokers more likely than never smokers to believe that e-cigarettes are cheaper than regular cigarettes.  REASONS top reasons: to reduce or quit smoking (58%), curiosity (19%), less smelly than cigarettes (19%). Perceived healthier (13%) can replace smoking cessation products (13%). |
| [40] | Baweja, Curci, Yingst, Veldheer, Hrabovsky, Wilson, Nichols, Eissenberg and Foulds [40] | n = 200 | U.S. | Mixed methods, self-report with open-ended questions (n = 200).  Types of user as defined by authors: ‘Current adult e-cig users (current user is not specified).’ All adult (18 years or older) e-cigarette users, median age 40.5 years. | PERCEPTIONS perceived as less harmful than smoking (36.5%). Safe way to use nicotine  REASONS health benefit (36.5%), smoking cessation (25%), vape quality (94%), battery life (82%) and liquids (59%). Starting e-cig use to quit tobacco soon (73.5%), e-cigs help quit smoking (93.5%). Pleasurable, improve sense of smell and taste, costs, routine maintenance, no odors, social environment. Device characteristics (design; ability to control voltage, simple to operate and maintain, durability, consistent performance of e-cig device and experience, taste and variety of flavors; throat hit, compatibilities of variety of e-cig components, machine quality, cost of e-cigarette device, battery life, tank size, safety features, easy availability of e-cigs, and the ability to customize liquids and coils), smoking cessation and reduced cigarette consumption; unexpected health effects; improved breathing; decreased cough, fewer sore throats; pleasure & smoking-related actions; less toxic than smoking tobacco; sense of smell and taste; less expensive than cigarettes; feasibility to use e-cigs; similar gestures or action of smoking cigarette; no unpleasant odors; taste and variety of flavors; safe for others or bystander with no second hand smoke; cravings; dental health. |
| [41] | Berg [41] | n = 1567 | U.S. | Cross-sectional (n = 1567)  Types of user as defined by authors: ‘Current smoker; smoked in the past 30 days. Former smoker; smoked more than 100 cigarettes in their lifetime but did not smoke in the past 30 days.’ 18-34 years old; REASONS for use among current e-cigarette users | PERCEPTIONS electronic cigarettes were perceived as one of the least harmful (41%), addictive and most socially acceptable.  REASONS less harmful than cigarettes (77%), do not smell (77%), smoking cessation (66%), costs (62%), weight management (6.6%) avoid smoking restrictions (25.2%), socially acceptable (48.7%), flavors (60.2%). |
| [42] | Biener and Hargraves [42] | n = 695 | U.S. | Mixed methods, self-report (n = 1374) and interviews (n = 695)  Types of user as defined by authors: ‘Level 3 = intensive users: used daily for at least 1 month. Level 2 = intermittent users: used more than once or twice but not daily for a month or more. Lever 1 = non-users or at most once or twice.’ Adult smokers (18 - 65 years old); motivation among e-cigarette users | REASONS to smoking cessation 52.6%, avoid smoking restriction 5.6%, social environment (16.1%), Cut back 4.5%, health benefit 8%.  PERCEPTIONS e-cig healthier than regular cigarettes (16.1%). |
| [43] | Biener, Song, Sutfin, Spangler and Wolfson [43] | n = 4,740 | U.S. | Self-report (n = 4740)  Types of user as defined by authors: ‘Current smokers: those who had at least smoked 100 cigarettes and either smoked every day or some days. Former smokers: those who had at least smoked 100 cigarettes but now smoked not at all. Never smokers: those who denied having smoked 100 cigarettes in their lifetime or never used any tobacco products.’ 18–35 years old; motivation asked among smokers | REASONS Curious 61.2% Current smokers; 59.1% former smokers; 77.3% Never smokers; Better for health than cigs 55.1% Current smokers; 42.7% Former smokers; 17.2% Never smokers; Friends use it 30.9% Current smokers; 28.1% Former smokers; 46.0% Never smokers; Can use in no-smoking areas 43.1% Current smokers; 33.8% Former smokers; Help to quit smoking 35.9% Current smokers; 40.1% Former smokers ; Cut down on smoking 41.1% Current smokers; 18.5% Former smokers; Doesn’t smell bad 42.7% Current smokers; 37.8% Former smokers; 25.9% Never smokers. |
| [44] | Bold, Kong, Cavallo, Camenga and Krishnan-Sarin [44] | n = 340 | U.S. | Longitudinal survey ever users, age 14–17.  Types of user as defined by authors: ‘Students were selected as ever  e-cigarette users if they responded  “yes” to the question “have you  ever tried an e-cigarette”. ’ | REASONS Interest: Curiosity, It is cool. Desirable attributes: Good flavors, Does not smell bad, and Hide from adults, Low cost. Social norms: Friends use, Parents/family use, Can use anywhere. Goal-directed: To quit smoking cigarettes, Healthier than cigarettes. |
| [45] | Brose, Brown, Hitchman and McNeill [45] | 2012 n = 4553, 2013 n = 1588, 2014 n = 1204 | UK | UK cohort study (n = 4553 in 2012, n = 1588 in 2013 & n = 1204 in 2014).  Types of user as defined by authors: ‘Current use: used e-cigarette daily, less than daily but at least once a week, less than weekly but at least once a month and less than monthly. Current smoker: Smoke cigarettes every day, not every day, or smoke tobacco of some kind. Ex-smoker: stopped smoking in the past year or more than a year ago.’ smokers and former smokers 18 years or older | PERCEPTIONS e-cigarettes perceived as less harmful than combustible cigarettes, but changed overtime (66.6%: 2012; 66.5%: 2013; 60.4%: 2014) |
| [46] | Brown, West, Beard, Michie, Shahab and McNeill [46] | n = 4117 | UK | Cross-sectional (n = 4117)  Types of user as defined by authors: ‘Current smokers: smoke every day, not every day or smoke tobacco of some kind but not cigarettes. Recent ex-smokers: stopped smoking completely in the last year.’ 18 years or older Current smokers and recent ex-smokers | PERCEPTIONS perceived as less harmful  Current smokers 67.6% believe less harmful; ex-smokers 63.2% less harmful  REASONS health benefit, cutting down & quitting (current uses), taste (ex-smokers)  Among Current users of e-cigarettes:  health benefit:82.6% current smokers, 83.5% recent ex-smokers; taste:24.4% current smokers, 39.4% recent ex-smokers; cutting down:83.0% current smokers, 78.9% recent ex-smokers; temporary abstinence:70.2% current smokers; 47.7% recent ex-smokers; quitting:82.8% current smokers, 84.4% recent ex-smokers |
| [47] | Chaffee, Gansky, Halpern-Felsher, Couch, Essex and Walsh [47] | n = 104 | U.S. | Cross-sectional (n = 104)  Types of user as defined by authors: ‘Current use: participants who had use e-cigarettes on one or more days in the past 30 days Ever use: participants who had hear of electronic cigarettes and ever used e-cigarettes, even once or twice.’ All male 13–18 years old; e-cigarette users and non-users | PERCEPTIONS believed to be likely (40%–75%): decreased athletic performance, trouble breathing, coughing, lung cancer, heart attack mouth cancer, mouth sores. Believed to be likely (40%–80%): upset your family, get into trouble, upset friends, bad breath, addictive, dental issues, harmful for others. Believed to be beneficial (15%–60%): relaxed, cool, fit in more, feel alert, and increased performance. Decreased athletic performance 57.3% never users, 28.0% ever users; Trouble catching your breath 54.7% never users, 19.2% ever users; Bad cough 53.2% never users, 23.9% ever users; Lung cancer 52.2% never users, 21.8% ever users; Heart attack 48.7% never users, 16.8% ever users; Mouth cancer 46.4% never users, 15.0% ever users; Mouth sores 45.5% never users, 20.8% ever users; Upset your family 68.2% never users, 38.2% ever users; Get into trouble 56.8% never users, 54.0% ever users; Upset your friends 50.2% never users, 18.4 % ever users; Bad breath 49.9% never users, 23.8% ever users; Become addicted 49.7% never users, 37.6% ever users; Brown teeth 41.5% never users, 17.2% ever users; Harm someone nearby 38.7 never users, 19.7% ever users.  REASONS Feel more relaxed 44.6% never users, 50.2% ever users; Look cool 39.2 % never users, 45.8 % ever users; Fit in more 33.2% never users, 36.4% ever users; Feel more alert 27.7% never users, 26.4% ever users; Increased athletic performance 18.0% never users, 19.2% ever users; |
| [48] | Cheney, Gowin and Wann [48] | n = 30 | U.S. | Qualitative interviews (n = 30) in the U.S. Aged 19–24 years old.  Types of user as defined by authors: ‘E-cigarette and dual users. (2) current use (within the past week) of e-cigarettes.’ | REASONS young adults: to continue e-cigarette use was that it kept them from smoking, to help them cope with stress, rewarding feelings of hand-to-mouth motions, form of entertainment, immediate health benefits, not smelling like smoke. Flavors filled a positive role as they were often a way to connect with other e-cigarette users, and a reason for continuing to use e-cigarettes, always many more to try, flavor made e-cig use more attractive than smoking. Dual use: help to cope with stress when exclusive use is not enough, bond with other smokers, convenience in a particular situation, influence young adult smokers with positive comments about e-cigs, family members introduced young adults to e-cigarettes, family member influence decision to start e-cigarette use, friends played a supportive role in initiation of e-cig use and continuation. It is perceived as socially acceptable behavior. |
| [49] | Coleman, Johnson, Tessman, Tworek, Alexander, Dickinson, Rath and Green [49] | n = 116 | U.S. | Qualitative research (focus groups n = 116).  Types of user as defined by authors: ‘Current use: use of an e-cigarette product in the past 30 days.’ 18–30 years old; current e-cig users | PERCEPTIONS persisted that e-cigarettes are less harmful. E-cigarettes a method to reduce or quit smoking, where smoking is not permitted, the perception of e-cigarettes as more socially acceptable, and the availability of a variety of flavors. Lack of knowledge about e-cigarettes ingredients affect in general and on health. Most tried e-cigarettes for the first time with friends, and overall, friends are positive.  REASONS ability to use as a smoking cessation method, avoid smoking restriction, socially acceptable, health benefit. However, not mimic smoking. |
| [50] | Dockrell, Morrison, Bauld and McNeill [50] | Structured interview, n = 1380 & self-report, n = 25,029 (2010 n = 12,587; 2012 n = 12,432) | UK | Mixed methods (structured interview, n = 1380 & self-report, n = 25,029)  Types of user as defined by authors: ‘smoking status: never-smoking, ex-smoking, occasional, and daily smoking. 18 years or older; both smokers (smokers’ survey) and general population (population survey).’ | PERCEPTIONS believed to aid smoking cessation efforts: 60% satisfy the desire to smoke, 55% Cut back on e-cigarettes, 53% believed it expensive, 51% help quit entirely, 39% believed it might not satisfy the desire to smoke, 71% perceived e-cigarettes as less harmful than combustible cigarettes. 28% considered them less harmful than Nicotine Replacement Therapy (NRT).  REASONS avoid smoking restrictions (43%), 35% to smoking cessation, 31% cut back on smoking. |
| [51] | Dutra and Glantz [51] | 2011 n = 17,353, 2012 n = 22,529 | U.S. | National Youth Tobacco Survey (n = 17,353 in 2011 & n = 22,529 in 2012): Types of user as defined by authors: ‘Experimenters; Have you ever tried cigarette smoking, even one or two puffs? 'Yes'. Ever smoker; 100 or more cigarettes (5 or more packs) of lifetime smoking. Current smoker; had smoked at least 100 cigarettes and smoked in the past 30 days.  Ever e-cigarette users: adolescents who responded ‘electronic cigarettes or e-cigarettes, such as Rayan or NJOY’ to the question ‘which of the following tobacco products have you ever tried, even just 1 time?’ Current e-cigarette users: those who responded ‘e-cigarettes’ to the question ‘during the past 30 days, which of the following tobacco products did you use on at least 1 day?’ Dual ever use: who have ever used e-cigarettes and ever smoked conventional cigarettes. Dual current use: who are currently using e-cigarettes and conventional cigarettes? 11–18 years old; Ever and current e-cigarette users.’ | REASONS Among current smokers, motives for ever e-cigarette use was related to quitting combustible tobacco use. smoking cessation |
| [52] | Eastwood, Dockrell, Arnott, Britton, Cheeseman, Jarvis and McNeill [52] | 2013 n = 2062, 2014 n = 1952 | UK | 11–18 years old. Types of user as defined by authors: population not stratified.  never smokers, former smokers, current smokers | PERCEPTIONS some perceived e-cigarettes to be less harmful to the user decreased significantly, from 73.4% in 2013 to 66.9% in 2014. Considered e-cigarettes to cause about the same level of harm to the user increased from 11.8% in 2013 to 18.2% in 2014. Believing them to be less harmful decreased from 78.5% in 2013 to 73.1% to in 2014, and similar levels of harm increased from 8.0% to 12.0%. |
| [53] | Etter [53] | n = 81 | France, Canada, Belgium & Switzerland | Self-report (n = 81)  Types of user as defined by authors: ‘daily user, non-daily user, former user, never used. Smoking status: daily, non-daily, former smoker, never smoker.’ 19–65 years old; Ever e-cigarette users | REASONS To quit smoking, for health benefits (e-cigarettes were perceived to be less toxic than tobacco), less expensive than regular cigarettes, can be smoked everywhere (including smoke-free places), to avoid disturbing other people, or producing environmental tobacco smoke or the smell of stale smoke, for the pleasure of smoking (including the pleasure of inhaling and smoking related actions), to reduce cigarette consumption, curious to test a *new* product, e-cigarettes taste and smell good, previously failed quit attempts, to get nicotine, unexpected health effects. |
| [54] | Etter and Bullen [54] | n = 3587 | 62% U.S. 14% France, 6% UK, 4% Switzerland, 3% Canada, 11% other countries | Self-report (n = 3587)  Types of user as defined by authors: ‘Daily users, occasional users (not daily), past users (used e-cigarettes in the past), and never users (never used e-cigarettes). 18 years or older e-cigarette users, smokers and non-smokers.’ | PERCEPTIONS less harmful than tobacco 83.5%  REASONS smoking cessation (76.8%), deal with cravings (79%), withdrawal symptoms (67%), costs (57.3%), avoid smoking restrictions (34%) (avoid going outside is 34.4%, avoid situations where you cannot smoke is 39.4%, social environment (to avoid bothering other people with tobacco smoke (44%)), avoid smoking restrictions (39%), cut down tobacco 28%), unable to stop using it (4%). Current smokers: helped them to reduce their smoking (92%). Former smokers: (96%) said that it helped them to quit smoking. Ever users: (89%) said that it was easy to abstain from smoking while using the e-cigarette |
| [55] | Faletau, Glover, Nosa and Pienaar [55] | n = 20 | New-Zealand, Auckland | Qualitative Research (Focus groups n = 20)  Types of user as defined by authors: Children aged 6–10 years; non-users | PERCEPTIONS children thought it looked cool, viewed is as an imitation cigarette, viewed as smoking. |
| [56] | Ford, MacKintosh, Bauld, Moodie and Hastings [56] | n = 20 | New-Zealand, Auckland | Youth Tobacco Policy Survey (n = 1205); cross-sectional  Types of user as defined by authors: ‘Regular smokers: at least one cigarette a week. Occasional smoker’s less than one cigarette a week. Never smokers: those who had never tried smoking, not even a puff or two. Ever smokers: regular smokers, occasional smokers, those who used to smoke and those who had tried smoking only once. 11–16 years old; ever (at least one cigarette a week), occasional smokers (less than one a week), those who used to smoke and those who had tried smoking only once, and never smokers.’ | PERCEPTIONS perceived harm differed for flavors, with candy and fruit flavors considered less harmful than tobacco flavors. Perception of flavor matching goal of using e-cigarettes (e.g., adult smoker giving up smoking would favor tobacco flavors). |
| [57] | Gowin, Cheney and Wann [57] | n = 30 | U.S. | Qualitative research (semi-structured individual interviews n = 30) with current e-cigarette users.  Types of user as defined by authors: ‘Current use of e-cigarettes (at least once per week). Young adults (19–31) who go straight to work (STW) from high school.’ | PERCEPTIONS e-cigarettes are healthier and a safer, e-cigarettes are cheaper, e-cigs are healthier and safer from themselves, it is a safer option than smoking, it is healthier and safer for others, no second hand smoke, nice smell, less harmful, environmental friendlier and reduces less waste.  REASONS healthier, safer, cheaper, safer for self and other, environmental friendlier than smoking |
| [58] | Hess, Antin, Annechino and Hunt [58] | n = 46 | U.S. | Qualitative research (focus groups n = 46). Age 18–25 years old.  Types of user as defined by authors: No type of user or frequency specified. | PERCEPTIONS utilitarian function and a social function. Social identity of e-cigarettes is described as different form the participants, described with an “us versus” them attitude. Utilitarian function neither as smoking reduction or cessation nor to minimize craving when smoking is prohibited. |
| [59] | Hilton, Weishaar, Sweeting, Trevisan and Katikireddi [59] | n = 86 | UK | Qualitative research (focus groups n = 86). Age 14–17 years old.  Types of user as defined by authors: ‘Smoking status: do you smoke cigarettes at all nowadays? And which statement describes you best: never tried, not even a puff or two; once had a puff or two, but never smoke now; do you sometimes smoke?  E-cigarette use: do you use e-cigarettes at all nowadays? And which statement describes you best: never tried, not even a puff or two; once had a puff or two, but never use e-cigarettes now; do you sometimes use e-cigarettes?’ | PERCEPTIONS potential health harms and unknown harmful ingredients. Unsure whether e-cigarettes are more or less addictive than conventional cigarettes.  REASONS great flavor, colors, fun tricks, fitting in, looking cool. |
| [60] | Huerta, Walker, Mullen, Johnson and Ford [60] | n = 3630 in 2012  n = 3185 in 2013  n = 3677 in 2014  Pooled n = 10,273 | U.S. | Health Information National Trends Survey (HINTS) 2012–2014.  Types of user as defined by authors: ‘Smoking status was defined in the same manner for this study and the original study, defining non-smokers as those who have smoked <100 cigarettes during their lifetime, current smokers as those who have  smoked >100 cigarettes and are still smoking every day or most days, and former smokers as those who have smoked >100 cigarettes in their lifetime and are not smoking now. U.S. adults (18>) smokers, former smokers and non-users.’ | PERCEPTIONS Perceived harm declined slightly from 2012 to 2014 (50.7% to 43.1%). Current and former smokers had higher odds of perceiving e-cigarettes as less harmful. |
| [61] | Kahr, Padgett, Shope, Griffin, Xie, Gonzalez, Levison, Mastrobattista, Abramovici, Northrup, Stotts, Aagaard and Suter [61] | n = 87 | U.S. | Qualitative research (focus group n = 87); Types of user as defined by authors: ‘pregnant women’s beliefs of e-cigarette users during pregnancy; use not specified.’ adult pregnant women; non-users. | PERCEPTIONS compared to combustible cigarettes, e-cigarettes were perceived as less harmful. However, e-cigarettes were not perceived safe during a pregnancy, damaging to baby, not safe during pregnancy, may be a smoking cessation tool, better alternative than regular cigarettes. Smoking causes health problems during pregnancy, smoking is selfish and irresponsible. Smoking is perceived as not acceptable during pregnancy; not as strong for e-cigarettes. Still risks and not taking care of her baby’s health if used. |
| [62] | Khoury, Manlhiot, Fan, Gibson, Stearne, Chahal, Dobbin and McCrindle [62] | n = 3312 | Canada | Self-report (cross-sectional survey n = 3312) with n = 238 adolescents who tried e-cigarettes at least once. (Age 14–15 years old).  Types of user as defined by authors: ‘E-cigarette use status: “Have you ever taken at least one puff from an electronic cigarette?” and “If yes, why did you try an e-cigarette?” (options: “a. It’s cool/fun/something new; b. For the buzz; c. To help me quit smoking; d. To help me smoke less; e. To help me when I’m not allowed to smoke”).  Smoking status: “Do you smoke now?” and “Think about the last 30 days. Did you smoke a cigarette, even a puff?’ | REASONS (among 238 adolescents ever users) cool/fun/new, for the buzz, helps to quit smoking, helps to smoke less, helps when not allowed to smoke. |
| [63] | Kim, Davis, Dohack and Clark [63] | n = 35 | U.S. | Qualitative research (focus groups, n = 35).  Types of user as defined by authors: ‘with adult e-cigarette users (18–65 years old) using e-cigarettes for at least two month prior to the study.  (1) those who were current users of both e-cigarettes and combustible cigarettes;  (2) those who were former combustible cigarette users; | PERCEPTIONS perceived as healthier, safer, and cleaner alternative compared to smoking. Respondents feel better; have more energy; breathe easier; cough less since using e-cigarettes. |
| [64] | Kinnunen, Ollila, Lindfors and Rimpela [64] | n = 10,233 | Finland | The 2013 and 2015 Adolescent Health and Lifestyle Survey, which is a cross-sectional postal survey.  Types of user as defined by authors: ‘Self-report (survey n = 10,233) among Finnish adolescents (12-, 14-, 16- and 18-year-olds obtained from the population register center). Tried e-cigarettes at least once.  E-cigarette use: “Have you ever tried electronic cigarettes? How many times altogether?” The options were: “I do not know what they are”, “No”, “I have tried once or twice”, “I have tried 20 times or less” and “I have tried more than 20 times”. Frequency was estimated in 2015: “Which one of the following alternatives best describes your current use of e-cigarettes?” with the options “I do not use e-cigarettes”, “I use e-cigarettes less than once a week”, “I use e-cigarettes once a week or more often, but not daily” and “I use e-cigarettes once a day or more often”.’ | REASONS to try something new, to quit smoking, friends use them, something new to try. |
| [65] | Kistler, Crutchfield, Sutfin, Ranney, Berman, Zarkin and Goldstein [65] | n = 34 | U.S. | Qualitative research (n = 34). Age 18–64 years old. Types of user as defined by authors: ‘Used e-cigarettes at least once.’ | REASONS reasons included: User Experience (The odor, feel, texture, appearance, taste, cloud chasing, and novelty); Social Acceptability (encouragement or acceptability to use e-cigs and to connect with others who vape *vice versa*  when there is stigmatization or no acceptability to use e-cigs); Cost; Health Risks/Benefits (health issues or benefits); Ease of Use (The difficulty or ease to manipulate or use an e-cig, availability of products, and setting in which it can be sued); Flavor (type of flavors, the mixing of flavors, the smell of flavors); Smoking Cessation Aid; Nicotine Content & the ability to control it; Modifiability; e-cigs Regulation; Dual use; Hobby use. |
| [66] | Lee, Lee and Cho [66] | n = 6655 | Korea | 2015 Korean Youth Risk Behavior Web-based Survey (n = 6655) age 13–18 years old ever e-cigarette users.  Types of user as defined by authors: ‘Ever conventional cigarette smokers “yes” to the question: “Have you ever tried a cigarette, even one puff, in your life?” Among ever-smokers, current conventional smokers: those who replied from “1 and 2 days” to “every day” for the question, “During the past 30 days, how many days did you smoke cigarettes, even one cigarette?”  Ever e-cigarette use: “yes” answer to the following question: “Have you ever tried e-cigarettes?” Current e-cigarette use: those who replied from “1 and 2 days” to “every day” to the question, “During the past 30 days, how many days did you use e- cigarettes?” The number of days that used e-cigarette was re-grouped into 0–2 days/month, 3–9 days/month, and 10 days/month.’ | REASONS Among ever e-cigarette users: curiosity (22.9%), belief that they were less harmful than conventional cigarettes (18.9%), to quit smoking (13.1%), to smoke indoors (10.7%). For infrequent e-cigarette users (<3 per month), curiosity was the most frequent reason for e-cigarette use (28.8%). For more frequent e-cigarette users (>10 per month), to quit smoking (21.0%) and indoor use (19.5%) were the most frequent reasons for e-cigarette use. The belief that e-cigarettes are less harmful was a common reason for use among both less (<3 per month) and more (>10 per month) frequent users of e-cigarettes (19.3% and 17.9%, respectively). |
| [67] | LeVault, Mueller-Luckey, Waters, Fogleman, Crumly and Jenkins [67] | n = 309 | U.S. | Based on the Minnesota Adult Tobacco Survey (2010) and the Brief Smoking consequences Questionnaire–Adult. Types of user as defined by authors: ‘n = 309; there were 235 current cigarette smokers consisting of 79 who smoked only cigarettes (smokers); 122 who used both cigarettes and e-cigarettes (dual users); and 34 former e-cigarette users. Only smokers and dual users were included in this analysis. 18 years or older.’ | REASONS reasons for dual use were to reduce or to quit smoking (79.5%). |
| [68] | Li, Bullen, Newcombe, Walker and Walton [68] | n = 840 | New-Zealand | The New Zealand Smoking Monitor (n = 840)  Types of user as defined by authors: ‘Sample of current smokers: who smoked at least one cigarette a month and who had not made a quit attempt lasting 24 hours or more in the past three months, and those who have made a quit attempt lasting 24 hours or longer in the past three months which may or may not have been sustained.  Use of e-cigarettes not specified.’ Current smokers and recent quitters 18 years or older | PERCEPTIONS One-third agreed that e-cigarettes were safer than tobacco cigarettes (n = 158) and agreed e-cigarettes could help people quit smoking (n = 162) |
| [69] | Li, Newcombe and Walton [69] | n = 2594 | New-Zealand | The New Zealand Smoking Monitor (n = 2594)  Types of user as defined by authors: ‘Ever use: Have you ever tried an electronic cigarette? Current use: which best describes how often you use an electronic cigarette now? At least once a day/at least once a week/at least once a month.  Current smoker’s non-attempter: smoked at least one cigarette a month and who had not made a quit attempt lasting 24 hours or more in the past three months. Recent quit attempters: those who have made a quit attempt lasting 24 hours or longer in the past three months which may or may not have been sustained.’ Current smokers and recent quitters 18 years or older. | REASONS 57.1% curiosity, 31.3% wanted to quit,8.4% alternative for tobacco cigarettes, 2.8% due to a recommendation, 2.5% safer, 2.4% avoid smoking restrictions, 1.2% costs (among ever users). 15.4% curiosity, 50.9% wanted to quit, 21.7% alternative for tobacco cigarettes, 0.8% due to a recommendation, 4.9% safer, 10.3% avoid smoking restrictions, 6.1% costs (among current users). |
| [70] | Lotrean [70] | n = 480 | ROMANIA | Cross-sectional data from Romania (n = 480) among students  Types of user as defined by authors: ‘Smokers: individuals who had smoked in the past month. Ex-smokers: those who had smoked in their lifetime but not in the past month. Non-smoker: those who had not smoked traditional cigarettes. Ever e-cigarette use: had tried at least once in lifetime. Students aged 19–24. 53.3% of the smokers, 25% of the ex-smokers and 5.5% of the non-smokers had tried e-cigarettes. Definition of use is not provided.’ | PERCEPTIONS E-cigarettes are less dangerous; overall 55.9%. Ever users; 62.3% smokers; 33.3% former smokers; 58.7% non-smokers; E-cigarettes can help smokers to quit; overall 66.4%. Ever users; 46.1% smokers; 70.8% former smokers; 79.4% non-smokers; E-cigarettes are used only by smokers; 48.9% overall.; 51.3% smokers; 50.0% former smokers; 46.8% non-smokers.  REASONS E-cigarettes are less dangerous 8% overall; 0% smokers; 50% former smokers; 0% non-smokers; To quit smoking 23.2% overall; 31.7% smokers; Curiosity 62.5% ever users; 65.9% smokers; 50.0% former smokers; 58.3% non-smokers; Other friends also tried e-cigarettes 23.2% overall; 25.6% smokers; 0% former smokers; 41.7% non-smokers. |
| [71] | Majeed, Stanton, Dube, Sterling, Burns and Eriksen [71] | n = 14 | U.S. | Qualitative research (focus groups (n= 14)). Types of user as defined by authors: ‘Current cigarette users (adults: 18 years or older) who ever used e-cigarettes. (self-identified as current smokers; had used e-cigarettes, even once).’ | REASONS for experimentation: curiosity, cravings, coolness, convenience, persuasive persons. Reasons for regular use: cravings, sensory experience, coolness, perceived reduced harm, convenience. |
| [72] | Majeed, Weaver, Gregory, Whitney, Slovic, Pechacek and Eriksen [72] | A total of 4170; 5717; and 6,051 respondents completed the 2012, 2014, and 2015 | U.S. | Tobacco Products and Risk PERCEPTIONS Surveys, cross-sectional, with U.S. adults (18>). Types of user as defined by authors: ‘Non-users, smokers, and former smokers. Current smokers were defined as adults who had smoked at least 100 cigarettes during their lifetime and reported currently smoking every day or some days. Former smokers were defined as adults who had smoked at least 100 cigarettes and responded not at all to the question about current smoking. Those who had not smoked at least 100 cigarettes in their lifetime were defined as never smokers.’ | PERCEPTIONS There is an increase perception that e-cigarettes are “about the same level of harm” as or to be “more harmful” than cigarettes. Uncertain about harm: decreased (47.8%) in 2012 to 29.5% in 2015. |
| [73] | Mark, Farquhar, Chisolm, Coleman-Cowger and Terplan [73] | n = 316 | U.S., University of Maryland Women’s Health Center | Self-report (n = 316) among pregnant women  Types of user as defined by authors: ‘Current use: past 30 days. Current smokers: smoked within the past 30 days.  Pregnant women (66.1 % reported having ever heard of e-cigarettes, 13% reported having any prior or current use of e-cigarettes (ever users), with 0.6% reporting current daily use); 18 years or older.’ | PERCEPTIONS less harmful for baby 43%, less harmful to self (45%), cheaper (31%), fashionable (18%), contain nicotine (57%), addictive (61%), contains tobacco (31%), not as bad for health (74%), taste better (54%), cut down (72%), avoid smoking restrictions (55%), smoking cessation tool (73%). |
| [74] | McKeganey and Dickson [74] | n = 650 | UK | Self-report (survey n = 650) among smokers. 18 years or older.  Types of user as defined by authors: ‘336 participants reported having tried/used e-cigarettes.’ No clear definitions provided. | REASONS to avoid smoking bans, flavors available, cheaper than cigarettes, less harmful than cigarettes, attractiveness of the device settings and specifications. Among smokers: switching to e-cig use was more enjoyable than smoking. If this expectation was nog met, it led to continued smoking. Also, it was important how the technology worked, how it looked when using e-cigs, how people reacted to them vaping, whether it was a poor substitute for smoking, if they felt embarrassed. |
| [75] | McQueen, Tower and Sumner [75] | n = 15 | U.S. | Qualitative research (interviews n = 15)  Types of user as defined by authors: ‘past year users, e-cigarette users who started using in the past year. 20–60 years old; e-cigarette users’ | REASONS alternative way of tobacco consumption, smoking cessation, costs, maintain weight, sense of taste, smell, ability to be physically active, health benefit (unexpected and experienced), Cut back on nicotine fix, not smelling of cigarette smoke, immediate effects outweigh potential long-term harm |
| [76] | Patel, Davis, Cox, Bradfield, King, Shafer, Caraballo and Bunnell [76] | n = 13,304 | U.S. | Internet surveys of U.S. adult conventional cigarette smokers and nonsmokers. n = 10,181 current cigarette smokers; n = 3123 nonsmokers aged 18 or older.  Types of user as defined by authors: ‘Current cigarette smokers = persons who had smoked at least 100 conventional cigarettes in their lifetime and currently smoked either “some days” or “every day” at the time of the survey.  Non-smokers were defined as persons who reported smoking “not at all” at the time of the survey, regardless of lifetime number of conventional cigarettes smoked.  Current e-cigarette users were defined as those who responded “every day” or “some days” to the question, “Do you now use e-cigarettes every day, some days, or not all?”’ | REASONS among current users: cessation/health (84.5%) (less harmful, reduce number of cigarettes, other friends use them too); consideration of others (less harmful, don’t smell); convenience (to avoid smoking bans), curiosity, flavors, costs, simulation of cigarettes.  Non-smokers: curiosity, tank users mentioned costs, cessation, and simulation of cigarette sensation. |
| [77] | Pearson, Richardson, Niaura, Vallone and Abrams [77] | n = 2649 (online study) & n = 3658 (cohort) | U.S. | Legacy Longitudinal Smoker Cohort (LLSC) (n = 3658) and national cohort (n = 2649)  Types of user as defined by authors: ‘in both surveys: Never smokers: having never smoked up to 100 cigarettes in their lives. Former smokers: having smoked 100 cigarettes or more in their lives but currently smoking not at all. Current smokers: having smoked in excess of 100 cigarettes in their lives and currently smoking every day or smoke days. 18–49 years old recent quitters and current smokers (n = 3658) and 18 years or older never, former, and current smokers (n = 2649).’ | PERCEPTIONS 70.6% of those aware in the online survey and 84.7% in the LLSC believed e-cigs are less harmful than combustible cigarettes. |
| [78] | Pepper, Emery, Ribisl, Rini and Brewer [78] | n =6607 | U.S. | Tobacco Control in a Rapidly Changing Media Environment (TCME) (n = 6607 current smokers)  Types of user as defined by authors: ‘Current use of e-cigarette: using them every day or some days. Ever use: tried e-cigarettes, even just one puff. Adult smokers; 18 years or older (mean age 44.2 years old)’ | PERCEPTIONS participants perceived e-cigarettes less likely to cause lung cancer, heart disease, and oral cancer compared to regular cigarettes. |
| [25] | Pepper, Ribisl and Brewer [25] | n = 1125 | U.S. | Self-report (survey n = 1125) age 13–17 years old  Types of user as defined by authors: ‘never smokers/e-cigarette users (89%), 4% current smokers and 5% current e-cigarette users. 3 categories of e-cigarette users: never users, ever users (used ≥1 time but not in the past 30 days), and current users (used ≥1 time in the past 30 days).’ | PERCEPTIONS Perceived fruit-flavored to be less harmful, and were more likely to try menthol, candy or fruit flavored e-cigarettes. |
| [79] | Pepper, Ribisl, Emery and Brewer [79] | n = 3878 | U.S. | Self-report (n = 3878)  Types of user as defined by authors: ‘Current use: using e-cigarettes either every day or some days. Ever use: trying or starting, even one puff. Adults (18 years or older) who ever tried e-cigarettes.’ | PERCEPTIONS Perceived as less harmful (29%) and less harmful to others (23%)  REASONS curiosity (53%), social environment (34%), to quit or cut back (30%), avoid smoking restriction (26%), unexpected benefits (42%), cravings (38%), affordable (28%), flavors (30%), routine maintenance (36%). |
| [80] | Peters, Meshack, Lin, Hill and Abughosh [80] | n = 47 | U.S. | Qualitative research (focus groups n = 47)  Types of user as defined by authors: age 15–17 years old male adolescent current e-cigarette users in Texas, U.S. | REASONS Aesthetics, accessibility, healthier than Cigarettes, Odorless, High Social Approval, Expeditious Consumption and Concealment, Safe High. |
| [81] | Pineiro, Correa, Simmons, Harrell, Menzie, Unrod, Meltzer and Brandon [81] | n = 1815 | U.S. | Self-report (n = 1815)  Types of user as defined by authors: ‘Dual users: participants reporting using tobacco cigarettes in the past 30 days, that is, users of both tobacco cigarettes and e-cigarettes. E-cigarettes users: reported smoking no tobacco cigarettes in the past month.’ 18 years or older; E-cigarette users | PERCEPTIONS taste, social environment, throat hit, weight control, addiction.  REASONS smoking cessation tool, health benefit, curiosity, due to family/friends, Cut back smoking, enjoy taste, deal with stress. Self-regulation. |
| [33] | Pokhrel, Herzog, Muranaka and Fagan [33] | n = 62 | U.S., Hawaii | Qualitative research (focus groups n = 62)  Types of user as defined by authors: ‘All participants’ current daily e-cigarette users.’ 18–35 years old; Current daily e-cigarette users and dual users | REASONS smoking cessation, health benefit, satisfaction, mimic smoking, cut back, avoid smoking restrictions, discreet (hiding use), hobby, social environment, costs |
| [82] | Pokhrel, Herzog, Muranaka, Regmi and Fagan [82] | N = 62 | U.S., Hawaii | Qualitative research (focus groups n = 62):  Types of user as defined by authors: ‘All participants’ current daily e-cigarette users. Former smokers: reported having smoked more than 100 cigarettes in the lifetime and non in the past 30 days. Current non-smokers: never smoked cigarettes or were former cigarette smokers. 18–35 years old; Current daily e-cigarette users and current dual users.’ Dual users, 18 years or older | REASONS Dual use: to help with cravings, situational use, places, when other substances are used, need of an e-cigarette substitute. Activities (working, before work out), places/situations (home, inside a vehicle, when you don’t want to smell), to avoid smoking restrictions |
| [32] | Rass, Pacek, Johnson and Johnson [32] | n = 350 | U.S. | Cross-sectional (n = 350)  Types of user as defined by authors: ‘all participants were dual users of e-cigarettes and tobacco cigarettes.’ Dual users, 18 years or older | PERCEPTIONS less harmful (57% much less, 30% some less), less harmful to others, less enjoyable than cigarettes, addictive, NRT perceived as equally harmful as e-cigarettes (59%). 30% not at all addictive, unsure about the dangers of e-cigarettes (22%), unsure about the dangers 22.0%  REASONS less harmful 64%; To cut down smoking tobacco 40%; Avoid smoking restrictions 45%; To quit smoking 34%; cravings 57%; less harmful to others 52%; experienced health benefits 30%; costs 27%; taste 22%; Other (e.g., prefer the smell, reduce stress) 3%; withdrawal 35%; focus 8%; I can’t stop using it 1%; With an e-cigarette, it is easier to just smoke one or a few puffs at a time rather than a whole cigarette 45%. |
| [83] | Richardson, Pearson, Xiao, Stalgaitis and Vallone [83] | n = 1487 | U.S. | Legacy Longitudinal Smoker Cohort (n = 1487)  Types of user as defined by authors: ‘Current smoker: those who reported smoking ‘every day’ or ‘some days’. Former smoker: those who reported smoking ‘not at all’. Current and former smokers 18–49 years old’ | PERCEPTIONS perceived as less harmful than combustible cigarettes overall (61.6% among smokers, 79.2% among ever users, and 65.4 % among aware but non users).  REASONS 55.3% cut back or smoking cessation, 38.1%, avoid smoking restrictions, feels like smoking (62.8%), with former smokers more likely than current smokers (35.5% vs. 16.8%). cost (59.6%), social environment (69.6%), no lingering odor (61.7%). avoid smoking bans (69.0%), with former smokers more likely than current smokers to cite this as a reason for use (92.6% vs. 65.4%). |
| [84] | Rutten, Blake, Agunwamba, Grana, Wilson, Ebbert, Okamoto and Leischow [84] | n = 2254 | U.S. | Cross-sectional (n = 2254)  Types of user as defined by authors: ‘Current smokers: had smoked 100 or more cigarettes during their life and smoke cigarettes currently. E-cigarette users: use e-cig on some days or every day. 18 years or older; Current smokers’ | REASONS Reduce health risks 51.9%; Quit smoking 58.4%; Reduce smoking 57.9%; Appealing flavor 14.7%; Not as strong, lighter 15.9%; Addicted to e-cigarettes 7.099%; Curious 16.0%; Stress reduction 11.9%; Cost less 24.5%; Can smoke indoors 46.8%; Less harmful to others 32.9%; |
| [85] | Saddleson, Kozlowski, Giovino, Goniewicz, Mahoney, Homish and Arora [85] | (n = 429) | U.S. | Cross-sectional (n = 1437) subsample (n = 429)  Types of user as defined by authors: ‘current use: past 30 days divided in current daily (all 30 days) and current non-daily (1 to 29 days of the previous 30 days) users. Discontinued e-cigarette user: ever used, but not in the previous 20 days. Never smokers: never tried a tobacco cigarette, not even a puff. Experimenters: have smoked less than 100 cigarettes in lifetime, and did not smoke any cigarettes in the past 30 days. Discontinued smokers: smoked 100 or more cigarettes in lifetime, but did not smoke any cigarettes in the past 30 days. Current smokers: have smoked at least 1 day out of the past 30 days. 18–23 years old, 29.8% e-cig ever users.’ | PERCEPTIONS less toxic (46.5%)  REASONS pleasure/enjoying use 57.9%, social environment (20.4%), alternative way of tobacco consumption (18.5%), smoking cessation (14.1%), cravings (13.6%), do not smell like smoke (39.2%), costs (24.9%), to try something new (71.6%), to try something new and to help control appetite (9.1%), I am addicted to the e-cig and because all other smoking cessation methods had failed (5.3%). |
| [86] | Saddleson, Kozlowski, Giovino, Hawk, Murphy, MacLean, Goniewicz, Homish, Wrotniak and Mahoney [86] | n = 1437 | U.S. | Self-report (n = 1437)  Types of user as defined by authors: ‘Current use: use on one or more days the past 30 days. Ever use: Have you ever tried or experimented with an e-cigarette, even one or two puffs? Never smoker: never tried a tobacco cigarette. Former smokers: smoked 100 or more cigarettes in lifetime, and have smoked 0 out of past 30 days. Experimenters: have ever tried a cigarette, have smoked less than 100 cigarettes in lifetime and have smoked 0 of the past 30 days. Current smoker: have smoked at least 1 day out of the past 30. 18–23 years old; general population; reports dual use for smokers’ | PERCEPTIONS E-cigarettes are less harmful than tobacco cigarettes: ever e-cig users (32.8%), current (16.3%), never (42.4%). |
| [87] | Schmidt, Reidmohr, Harwell and Helgerson [87] | n = 5067 | U.S. | Adult Tobacco Survey (self-report) cross sectional data of noninstitutionalized Montana adults. Types of user as defined by authors: “Have you ever used an electronic cigarette, even just one time in your entire life?” 🡪 ever use  “Do you now use electronic  cigarettes every day, some days 🡪 current e-cigarette users.  Adult (18+) ever users of e-cigarettes (who answered the questions regarding REASONS)’ | REASONS curiosity, to quit, less harmful, less disturbing, to avoid smoking bans, taste, costs. |
| [88] | Sherratt, Marcus, Robinson, Newson and Field [88] | n = 319 | UK | Cross-sectional (n = 319)  Types of user as defined by authors: ‘Current smokers: smoked one or more cigarettes within the past week. Recent former smokers: did not smoke one or more cigarettes within the past week. Ever e-cigarette use: have ever used an electronic cigarette. Current e-cigarette use: having used an e-cigarette within the past month. Former e-cig user: used within the past 1-6 months or more than 6 months ago. current smokers: Stop smoking service, 18–60 years old’ | PERCEPTIONS 48.2% perceived e-cig as less harmful than tobacco. 38.8% felt uncertain about e-cig safer than tobacco. Current users viewed e-cig as less harmful than former or never users. |
| [89] | Sherratt, Newson, Marcus, Field and Robinson [89] | n = 20 | UK | Qualitative interviews (n = 20).  Types of user as defined by authors: ‘With participants of Stop Smoking Services in north-west England. Both individuals who had tried e-cigarettes (n = 6) and those who had not (n = 14). Median age was 51.5 years (range 25–59). Recent former smokers: they had not smoked within at least the last 7 days)’ | PERCEPTIONS e-cigarette users perceive e-cigarettes as safer than smokers. E-cigarettes were perceived as an effective smoking cessation aid. Reduce cravings and helps to sustain abstinent from tobacco. |
| [90] | Simmons, Quinn, Harrell, Meltzer, Correa, Unrod and Brandon [90] | n = 31 | U.S. | Qualitative research (focus groups n = 31). With e-cigarette users.  Types of user as defined by authors: ‘(1) ≥ 18 years old; (2) had smoked cigarettes daily for at least 1 year; and (3) had used e-cigarettes in the past 30 days.’ | PERCEPTIONS perceived health benefits.  REASONS to quit smoking initially. Interest and satisfaction from experimenting with several aspects of e-cigarette devices. |
| [91] | Soule, Rosas and Nasim [91] | n = 108 | U.S. | Concept mapping (CM) to characterize and describe adults’ REASONS for using ECIGs.  Types of user as defined by authors: ‘A total of 108 adults completed a multi-module online CM study that consisted of brainstorming statements. E-cigarette use was described as past 30-day use. 18 years or older and used e-cigarettes in the past month.’ | REASONS Cessation Methods, perceived health benefits, private regard, convenience, conscientiousness, and pleasurable effects, perceived agency, therapeutic, hobby, and social impacts. |
| [92] | Suris, Berchtold and Akre [92] | n = 621 (248 e-cigarette users) | Switzerland | Longitudinal research (self-report (n = 621)  Types of user as defined by authors: ‘Current smoker: smoking at least weekly. Experimenters: only used once. Adolescent (14–16 years old). Users (experimenters and regular users) were asked about REASONs’ | REASONS Curiosity 93.1% (experimenters)–76.8% (users); avoid smoking restrictions 3.2% (experimenters)–14.4% (users); To reduce smoking 3.6% (experimenters)–10.4% (users); To do like my friends 5.1% (experimenters)–6.0% (users); To smoking cessation 0.0% (experimenters)–3.3% (users). |
| [93] | Tan and Bigman [93] | n = 3630 | U.S. | Health Information National Trends Survey (n = 3630); cross-sectional  Types of user as defined by authors: ‘Non-smoker: respondents who have never smoked up to 100 cigarettes in their lives. Former smokers: Those who smoked at least 100 cigarettes in their lives but were currently not smoking at all. Current smokers: those who smoked at least 100 cigarettes in their lives and were smoking daily or on some days. 18 years or older; general population’ | PERCEPTIONS decline in harm perception among current: believed e-cigarettes are less harmful than smoking (84.7% in 2010 to 65.0% in 2014). Believe e-cigarette as less harmful 49.5%former smokers, 45.9 % non-smokers, and 65.0% current smokers. |
| [94] | Tan, Lee and Bigman [94] | n = 527 | U.S. | Self-report (n = 527)  Types of user as defined by authors: ‘Ever users: comprising those tried but not in the past 30 days, and those used in the past 30 days, even just one time.’ 18–87 years old; general population. | PERCEPTIONS Harmful to others 42.8% Non-users; 26.2% ever-users; addictive 52.5% Non-users; 40.9% ever-users; Gateway effect 55.1% Non-users; 43.1% ever-users; Socially acceptable 61.6% Non-users; 38.5% ever-users; Less harmful than smoking 32.4% Non-users; 56.9% ever-users; Smoking cessation 31.9% Non-users; 52.3% ever-users; Less harmful to others 38.9% Non-users; 57.6% ever-users; make smoking look acceptable to youth never smokers;35.7%, former smokers 46.7%, current smokers 36.1% |
| [95] | Trumbo and Harper [95] | n = 244 | New-Zealand | Cross-sectional (n = 244)  Types of user as defined by authors: Ever tried and regular use not specified; 19–22 years old; not specified | PERCEPTIONS students found it socially acceptable to use e-cigarettes in public places. Overall negative attitude, pressure to use an e-cigarette |
| [96] | [96] | n = 3241 | U.S. | Longitudinal research (survey 7-monght follow-up).  Types of user as defined by authors: ‘Ever use of e-cigarettes (n=2476 are included in the analysis). “Have you ever used e-cigarettes, electronic, or vapor cigarettes?” adult ever users of e-cigarettes (18+)’ | REASONS to quit, to replace tobacco cigarettes, to cut down, to avoid smoking bans, curiosity, deal with cravings, less harmful, recommendation, costs |
| [97] | Wackowski, Bover Manderski and Delnevo [97] | n = 519 | U.S. | Cross-sectional (n = 519)  Types of user as defined by authors: ‘current smokers: have ever smoked 100 cigarettes and now smoke some days or every day. Current e-cigarette users: those who had used e-cigarettes in the past 30 days. Former e-cigarette users/triers: those who had ever tried e-cigarettes but not used them in the past 30 days. Current Cigarette smokers 18 years or older.’ | PERCEPTIONS 59.9% of smokers believed e-cigarettes are less harmful. More prevalent among current e-cig users (82.8%) than former (63.9%) or never (48.6%) |
| [98] | Wackowski, Bover Manderski, Delnevo, Giovenco and Lewis [98] | n = 509 | U.S. | Self-report (survey n = 509) among adult (18 years or older)  Types of user as defined by authors: ‘current smokers defined as having ever smoked 100 cigarettes and now smoking “some days” or “everyday” in the U.S. Those smokers who had also used e-cigarettes in the past 30 days as “current e-cigarette users/triers”. Those who had ever tried e-cigarettes but not used them in the past 30 days as “former e-cigarette users/triers”.’ | REASONS believed it was less harmful than regular cigarettes (77.2%), out of curiosity (76.5%), as a way to cut down on smoking (72.7%), cigarette smoking cessation (64.9%), to use in places where they can’t smoke (66.7%). Current e-cigarette users/triers reported more often cutting down on smoking, saving money and consideration for others as reasons for trying e-cigarettes. Curiosity was the main reason for trying e-cigarettes among former users/triers (77.1%). |
| [99] | Wang, Li, Jiang, Chu, Kwong, Lai and Lam [99] | n = 1307 | Hong Kong | Cross-sectional (n = 1307)  Types of user as defined by authors: ‘Daily smokers; 4 ppm or above exhaled carbon monoxide. Ever use: ever used, even a single puff, age 18 years or older. 18 years or older; daily smokers’ | PERCEPTIONS 11.0% perceived is as an effective smoking cessation tool. 74.1% (users) and 91.2% (non-users) did not think of them as effective for smoking cessation. |
| [100] | White, Li, Newcombe and Walton [100] | 2012 n = 3127, 2014 n = 2919 | New-Zealand | Youth Insights Survey (2012: n = 3127; 2014: n = 2919); cohort  Types of user as defined by authors: ‘Current smokers; smoked at least once a month or more often. Infrequent smokers; less often than once a month. Ex-smokers; had smoked a cigarette, but no longer smoke. Ever use: have ever tried electronic cigarettes. 14–15 years old; not stratified (ever users are asked reasons why)’ | REASONS curiosity (64.5%), recommended by someone (24.2%), safer than tobacco (27.8%), avoid smoking restrictions (16.3%), cut back on cigarettes (18.4%), smoking cessation (16.6%), costs (not specified). Curiosity: infrequent smokers (67.1%) and ex-smokers (62.4%). Harm reduction: 42.5% infrequent smokers having first tried e-cigarettes for this reason. |
| [101] | Yong, Borland, Balmford, Hitchman, Cummings, Driezen and Thompson [101] | n = 2105 | Australia & UK | International tobacco control four country ITC project, Australia & UK 2013 (n = 2105). Longitudinal data.  Types of user as defined by authors: ‘E-cigarette use: “Have you ever tried an electronic cigarette?” Those who had tried: “How often, if at all, do you currently use an electronic cigarette?” with the response options “Daily, Less than daily, Less than weekly, Less than monthly or Not at all”.’ | PERCEPTIONS Australia: a lot less harmful compared to conventional cigarettes 36% of the smokers. UK: a lot less harmful compared to conventional cigarettes 57.6% of the smokers. |
| [102] | Zhu, Gamst, Lee, Cummins, Yin and Zoref [102] | n = 10,041 | U.S. | Cross-sectional n = 10,041  Types of user as defined by authors: ‘Current smoker; had at least smoked 100 cigarettes in their lifetime and answered the question 'Do you currently smoke cigarettes every day, some days, or not at all?' with every day or some days. Former smokers; those who smoked at least 100 cigarettes in their lifetime and answered ‘not at all’. Non-smoker: those who had not smoked 100 cigarettes in their lifetime. Ever use: those who have ever tried an e-cigarette. Current user: those who had used e-cigarettes in the last 30 days. 18 years or older; general sample, reasons among e-cig users’ | REASONS Safer than cigarettes 49.9%; Cheaper than cigarettes 30.3%; Easy to use when I can't smoke 44.8%; To try to quit smoking cigarettes 54.9%; Just because 68.3% |
